# EpiFusion: Joint inference of the effective reproduction number by integrating phylodynamic and epidemiological modelling with particle filtering

**DOI:** 10.1371/journal.pcbi.1012528

**Published:** 2024-11-11

**Authors:** Ciara Judge, Timothy Vaughan, Timothy Russell, Sam Abbott, Louis du Plessis, Tanja Stadler, Oliver Brady, Sarah Hill

**Affiliations:** 1 Department of Infectious Disease Epidemiology and Dynamics, Faculty of Epidemiology and Public Health, London School of Hygiene and Tropical Medicine, United Kingdom; 2 Centre for Mathematical Modelling of Infectious Diseases, London School of Hygiene and Tropical Medicine, United Kingdom; 3 Department of Pathobiology and Population Sciences, Royal Veterinary College, United Kingdom; 4 Department of Biosystems Science and Engineering, ETH Zurich, Basel, Switzerland; 5 Swiss Institute of Bioinformatics, Lausanne, Switzerland; Ecole Normale Superieure, FRANCE

## Abstract

Accurately estimating the effective reproduction number (R_t_) of a circulating pathogen is a fundamental challenge in the study of infectious disease. The fields of epidemiology and pathogen phylodynamics both share this goal, but to date, methodologies and data employed by each remain largely distinct. Here we present EpiFusion: a joint approach that can be used to harness the complementary strengths of each field to improve estimation of outbreak dynamics for large and poorly sampled epidemics, such as arboviral or respiratory virus outbreaks, and validate it for retrospective analysis. We propose a model of R_t_ that estimates outbreak trajectories conditional upon both phylodynamic (time-scaled trees estimated from genetic sequences) and epidemiological (case incidence) data. We simulate stochastic outbreak trajectories that are weighted according to epidemiological and phylodynamic observation models and fit using particle Markov Chain Monte Carlo. To assess performance, we test EpiFusion on simulated outbreaks in which transmission and/or surveillance rapidly changes and find that using EpiFusion to combine epidemiological and phylodynamic data maintains accuracy and increases certainty in trajectory and R_t_ estimates, compared to when each data type is used alone. We benchmark EpiFusion’s performance against existing methods to estimate R_t_ and demonstrate advances in speed and accuracy. Importantly, our approach scales efficiently with dataset size. Finally, we apply our model to estimate R_t_ during the 2014 Ebola outbreak in Sierra Leone. EpiFusion is designed to accommodate future extensions that will improve its utility, such as explicitly modelling population structure, accommodations for phylogenetic uncertainty, and the ability to weight the contributions of genomic or case incidence to the inference.

## Author summary

Understanding infectious disease spread is fundamental to protecting public health, but can be challenging as disease spread is a phenomenon that cannot be directly observed. So, epidemiologists use data in conjunction with mathematical models to estimate disease dynamics. Often, combinations of different models and data can be used to answer the same questions–for example ‘traditional’ epidemiology commonly uses case incidence data (the number of people who have tested positive for a disease during a certain time period) whereas phylodynamic models use pathogen genomic sequence data and our knowledge of the way their genomes evolve to model disease population dynamics. Each of these approaches have strengths and limitations, and data of each type can be sparse or biased, particularly during rapidly developing outbreaks or in countries with poor pathogen surveillance. An increasing number of approaches attempt to fix this problem by incorporating diverse concepts and data types together in their models. We aim to contribute to this movement by introducing EpiFusion, a modelling framework that improves the efficiency and precision at which we can monitor important changes in pathogen transmission (specifically, in the effective reproduction number). EpiFusion uses particle filtering to simulate epidemic trajectories over time and weight their likelihood according to both case incidence data and a phylogenetic tree using separate observation models, resulting in the inference of trajectories in agreement with both sets of data. Improvements in our ability to accurately and confidently model pathogen spread help us to respond to infectious disease outbreaks and improve public health.

## Introduction

The effective reproduction number (R_t_) is a helpful epidemiological parameter for characterising disease transmission. R_t_ refers to the time-varying average number of secondary infections resulting from a primary infected individual and can vary due to factors such as population immunity, human behaviour, or changes in pathogen infectiousness. Retrospective modelling of how R_t_ varies over the course of an outbreak allows for evaluation of policy and intervention efficacy [1–4], and quantifying how different factors contribute to R_t_ can inform outbreak preparedness planning by providing the basis for modelling spread under different scenarios [5]. Classical epidemiology [3] and phylodynamics [4] often aim to infer R_t_ but use distinct methodologies and data to achieve this goal. Both fields face similar but non-overlapping obstacles in terms of data availability, reliability, and bias [6–9]. We investigate an approach to estimate R_t_ that reduces this uncertainty through linking principles of phylodynamic and epidemiological modelling using particle Markov Chain Monte Carlo (pMCMC) [10] which is scalable to large datasets.

Phylodynamic approaches allow estimation of the genealogical history of genome-sequenced sampled viruses and can therefore inform about disease spread that occurred prior to the first identified case. Phylogenetic trees frequently capture unusual population dynamics [11] that are not normally detectable using case data alone, such as long-range virus lineage movements, importations or growth in the dominance of specific variants. However, a central challenge for phylodynamics is that genomic data sampling density can be low or spatiotemporally biased relative to infection occurrence [12]. Furthermore, R_t_ has thus far been commonly estimated as a piecewise constant function that rarely has sufficient temporal resolution to be useful for public health decision making [13], with some exceptions [14].

Conversely, epidemiological models of R_t_ use case data that are often more spatiotemporally consistently sampled than genomic data, and usually have greater flexibility than phylodynamic models to accommodate additional information such as climatic or human movement data [15–18]. However, case data can be easily biased by changes in case definitions or reporting practices [7,19] which can cause artificial fluctuations in R_t_ estimates. Disease dynamics can only be examined once individuals with infections are detected, which may not occur until long after a pathogen starts to spread (whereas phylogenetic tree data can be used to reconstruct past pathogen dynamics prior to the sampling date of the earliest genome). Furthermore, viruses that can cause similar clinical symptoms (such as Zika, chikungunya and dengue viruses [20,21]) can be easily misreported where specific molecular or serological testing is not conducted. This can result in the inferred R_t_ capturing the average population dynamics of multiple cocirculating pathogens, which is then less useful to inform disease-specific control measures such as vaccination programs [22–24].

As a result of these limitations and strengths, phylodynamic and epidemiological approaches may vary in their effectiveness at different stages of an outbreak [12]. Approaches that combine principles and data from both phylodynamic and epidemiological models could improve our ability to estimate R_t_, by taking advantage of the complementary strengths of each field.

Early attempts to use both phylodynamics and epidemiology to estimate disease dynamics typically employed a ‘corroborate or contradict’ strategy, where methods and data native to each field were used separately to address the same research question [25–27]. Alternatively, methods from each field have sometimes been used to address different research questions in the same study [28]. Recently, attempts have been made to develop joint inference approaches that use both phylodynamic (dated genomic sequence) and epidemiological (case incidence) data as input to a single model [29–34]. Many of these attempts have built on the principle of the particle filter [10]. Particle filtering is a sequential Monte Carlo approach that aims to approximate the posterior distribution of a state variable in a stochastic process (in this case, an epidemic trajectory). Particles move through a hidden Markov Model (the process model) and are weighted by their likelihood according to the data (the observation model). They can then be resampled according to their weights, resulting in the propagation of particles with estimated states consistent with the data under an observation model. The use of particle filtering is arguably the most straight-forward method to directly link epidemiological and phylodynamic models, as the resampling of particles through time allows the genealogical and epidemiological data to jointly influence the particle states during the state-simulation process.

Particle filtering is well established for use with epidemiological case incidence data, and there are many existing implementations of particle filtering in epidemiological modelling [35,36]. More recently, appropriate particle filtering approaches have been developed that can use genealogies obtained from sequence data. Rasmussen et. al first proposed a joint inference approach consisting of a common process model and separate observation models for a genealogy and case incidence data [30]. This methodology was later extended to allow fitting of epidemiological models that incorporate simple population structure [31], and was also used as the basis of an approach for inferring transmission heterogeneity [37]. These models were all reliant on coalescent-based phylodynamic methods and assumed independence between case incidence and events in the phylogenetic tree [38]. In 2019 Vaughan et. al proposed a method ‘EpiInf’, that enable the use of birth-death phylodynamic methods within a particle filter to infer epidemic trajectories through time [32]. EpiInf derived a phylodynamic likelihood that explicitly models case incidence data as ‘unsequenced observations’ within the phylodynamic observation model as ‘events’ on the tree, thus overcoming the independence assumption made in earlier approaches. However, this latter approach quickly becomes intractable as the number of sequences or cases increases (even when using a tau-leaping approximation [39]). It also greatly limits the possible complexity that could be obtained using a separate epidemiological observation model, which could feasibly incorporate diverse data sources (e.g. climate or human movement data). Conversely TimTam, proposed by Zarebski et al. in 2022 [29,40], is a (non-particle filtering) birth-death phylogenetic approach that can integrate case incidence and genomic sequence data in a computationally tractable way by approximation of the birth-death observation model density [41,42], while also eliminating the assumption of independence between tree and occurrence events. However, while it is possible to infer prevalence at user-specified times and R_t_ in piecewise constant intervals, it is not practical to infer continuous (here we use the term ‘continuous’ to refer to a fine grid size of a single day) epidemic trajectories with this model, which limits its ability to detect transmission fluctuations at a higher temporal resolution.

We develop a new approach, EpiFusion, that extends existing implementations that employ particle filtering or pMCMC [30,32] to reconstruct epidemic trajectories using case incidence and a phylogenetic tree either individually or together, while making the assumption that the tree and case trajectory are independent of each other. Our proposed approach improves on the limitations of previous methods by *(i)* introducing a birth-death based phylodynamic likelihood to a dual observation model structure *(ii)* making improvements in computational efficiency and *(iii)* allowing epidemic trajectories to be inferred in greater temporal resolution.

## Methods

### Theory

**Fig 1:**
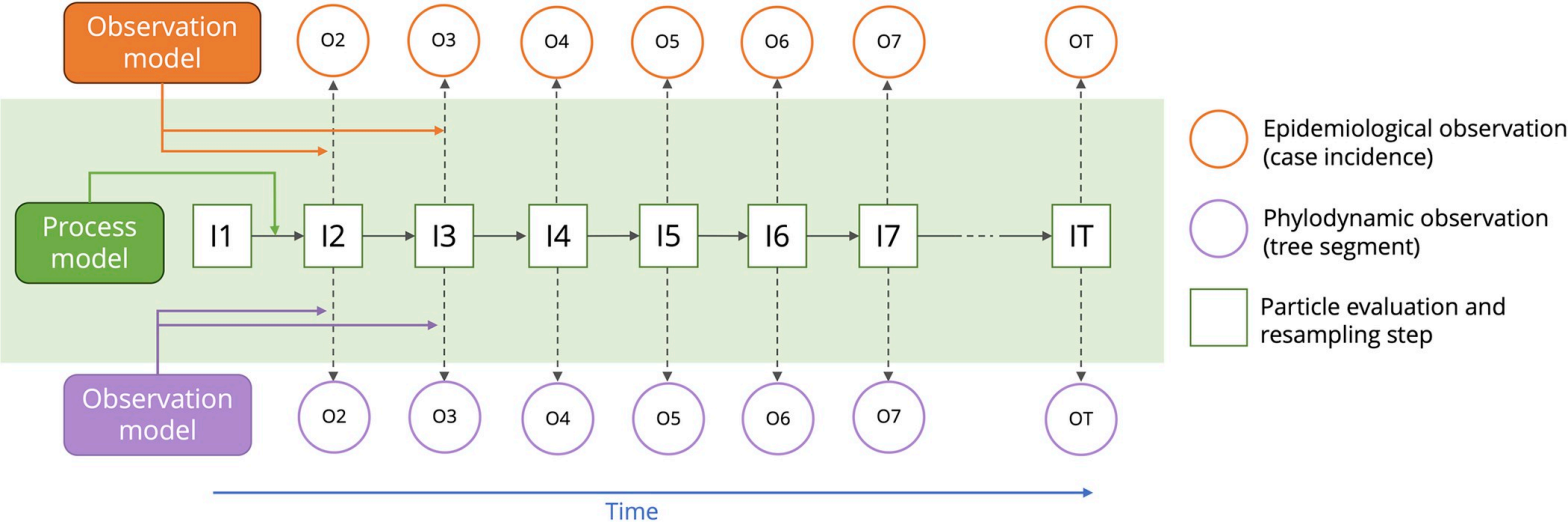
EpiFusion particle filter structure, with the particle states per unit time (green outlined boxes) driven by the parameters of the process model, evaluated at resampling steps by epidemiological and phylodynamic observation models against case incidence and phylogenetic tree segments respectively per unit time (orange and purple circles). All models in this manuscript use daily time units.

We adopt an overall structure based on the ‘common process model–dual observation model’ structure *(Fig 1)* used by Rasmussen et. al [30] and validated by many particle filtering methods outside of the context of infectious disease [43,44]. The data inputs (‘observations’ in Fig 1) to this model are case incidence, a time-scaled phylogenetic tree constructed from virus genomic sequences, or both data types together. The hidden particle states are the true number of individuals infected ‘*I*’ and any particle specific parameters.

#### Process model

We use the term ‘process model’ to define how particle states are incremented between resampling steps in the particle filter. *n* particles model the number of infected individuals (*I*) in discrete daily intervals driven by a process model that assumes independent Poisson-distributed infection and recovery counts *(Eq* *1)*.


$$I_{t}=I_{t-1}+Pois(\beta_{t}I_{t-1})-Pois(\gamma_{t}I_{t-1})$$ (1)

We have implemented this daily discretisation rather than modelling each infection trajectory event individually in completely continuous time to improve computational efficiency. Transmission dynamics are captured by modelling the change in the infection rate *β* and/or recovery rate *γ* over time (see Table 1B legend). R_t_ can be derived from the process model using the formula $R_{t}=\frac{\beta_{t}}{\gamma_{t}}$.

**Table 1:** A. Explanation of the data points used by EpiFusion; B. the key parameters of the EpiFusion particle filter. Gamma, phi and psi are fit by MCMC, either as constant values over time or in epochs by either fixing or fitting change times and interval values. Beta must vary over time and can either be fit using (i) a random walk within the particle filter, (ii) linear splines within the particle filter, (iii) MCMC fitting in epochs by fixing or fitting change times and interval values, or (iv) MCMC fitting the parameters of a logistic function which defines beta over time; C. Other key terms in the EpiFusion particle filtering algorithm, in order of appearance in the text.

**A. Model Data**		
Name	*Symbol*	*Details*
Case Incidence Data	*c*_*t*_	Number of new observed disease cases with symptom onset in the interval between the previous case incidence data point. Each observation is associated with a single day (*t*) but represents the aggregation of cases between the last case incidence data point and day *t*
Phylogenetic Tree Segment	*g*_*t*_	A daily segment of a phylogenetic tree. Key attributes of each segment are the number of lineages over time, number of birth events, and number of sampling events. The tree can be provided to EpiFusion in its original form, and the segments *g*_*t*_ are generated by the program itself.
**B. Key Model Parameters**		
Name	*Symbol*	*Details*
Beta	*β*	The daily rate at which each infected person causes new infections. Must vary over time.
Gamma	*γ*	The daily rate at which each infected person becomes uninfectious. Can vary over time.
Phi	*φ*	The daily rate at which each infected person is sampled as a ‘case’ for case incidence data. Can vary over time.
Psi	*ψ*	The daily rate at which each infected person results in a sequenced pathogen sample. Can vary over time.
**C. Other terms**		
Name	*Symbol*	*Details*
Modelled daily case incidence	*ρ*_*t*_	The modelled number of individuals who are identified as cases with symptom onset on a given day *t*. Given by multiplying the number infected at time *t* by the sampling rate at time *t*: *I*_*t*_*φ*_*t*_
Modelled case incidence over an interval	*ρ*_*interval*_	A sum of *ρ*_*t*_ over a time interval that can be compared to observed case incidence over the same time interval by the epidemiological observation model.
Number of birth events	*b*_*t*_	Number of birth (branching) events in a tree segment *g*_*t*_
Number of sampling events	*s*_*t*_	Number of sampling events (tree leaves) in a tree segment *g*_*t*_
Number of lineages	*l*_*t*_	Number of lineages (branches) in the tree at the beginning of tree segment *g*_*t*_
Number of particles	*n*	The number of particles used in the particle filter
Particle weight	*ω*_*t*_	The particle weight at time *t*

#### Observation models

At each resampling step, the particle states are evaluated against epidemiological and phylogenetic data using individual ‘observation models’; that is, models that define the weights (*ω*) of each particle state according to each dataset.

The provided epidemiological data *c*_*t*_, represents the number of reported cases with symptom onset between regular intervals. As the particle infection trajectory is simulated through time, it ‘emits’ daily positive cases *ρ*_*t*_ at a rate *I*_*t*_*φ*_*t*_. These positive cases are summed for the days in the interval between case incidence observations. When *t* is a day with observed data, then this total can be evaluated against the total summed emitted cases in the corresponding interval *ρ*_*interval*_ *(Table 1C*), using the epidemiological observation model (*Eq 2*). This is not needed when case incidence is provided in daily intervals, in which case *ρ*_*t*_ can be directly compared to *c*_*t*_. Examples of the fit of *ρ*_*interval*_ to corresponding case incidence data points in practice are available in *S1 Fig.* This process gives the ‘epidemiological weight’ of the particle given the case incidence data *(ω*^*c*^_*t*_, *Eq* *2)*. Currently users may choose between a Poisson probability density function (*Eq* *2A*) and a negative binomial probability density function (*Eq* *2Bb*) with an overdispersion parameter *k* for the epidemiological weight. Here we use a Poisson model as there is no overdispersion in the simulated datasets used for validation.


$${\omega^{c}}_{t}=P(c_{t}|I_{interval},\varphi_{interval})=\frac{{\rho_{interval}}^{c_{t}}∙e^{-\rho_{interval}}}{c_{t}!}$$ (2A)

$${\omega^{c}}_{t}=P(c_{t}|I_{interval},\varphi_{interval},k)=\frac{\rho_{interval}∙e^{c_{t}}}{(k+\rho_{interval})e^{\left(k+c_{t}\right)}}$$ (2B)

The particle weight given the phylodynamic data (a one-day segment of a time-scaled phylogenetic tree; *g*_*t*_) is a daily discretisation of that which was derived by Vaughan et. al for EpiInf [32] (*Eq* *3*). This is the sum (in log space) of the probabilities of the observed events (number of observed infections of new individuals *b*_*t*_; number of sampling events *s*_*t*_) on the tree segment and the exponentially distributed waiting times for events that were not observed (infections with rate *β*_*t*_*I*_*t*−1_ and genomic samplings of infected individuals with rate *ψI*_*t*−1_).


$${\omega^{g}}_{t}=exp\left(b_{t}log(\frac{2\beta_{t}}{I_{t}-1})+s_{t}log\psi_{t}-({\psi_{t}I}_{t-1}+\beta_{t}I_{t-1})\right)$$ (3)

We implement an importance sampling strategy to prevent trajectory events that are impossible given the tree structure, for example recovery events that result in fewer individuals being infected than there are lineages in the tree (S*1 Text*).

During resampling, the particles are weighted (*ω*_*t*_) by the product of their phylodynamic and epidemiological weights (*Eq* *4*), thus facilitating the propagation of particles that are consistent with both the phylogenetic and epidemiological data.


$$\omega_{t}={\omega^{c}}_{t}{\omega^{g}}_{t}$$ (4)

#### Fitting with MCMC

Following completion of the particle filter, the overall likelihood of each estimated trajectory across the whole outbreak consists of the product of the average particle weights at each resampling step (*Eq* *5*). This is therefore the likelihood of a particle trajectory sampled from the surviving particles given the epidemiological and phylogenetic data, and the parameter set of the particle filter *θ* which can be concurrently fit using MCMC.


$$P(traj|g,c,\theta )=\prod^{T}\frac{\sum^{n}{\omega^{c}}_{t}{\omega^{g}}_{t}}{n}$$ (5)

This model is fit using Metropolis-Hastings MCMC sampling, deriving posterior samples of the number of infected individuals over time, and the rates *β*,*γ*,*φ* and *ψ*. Options are available for defining and fitting time-varying rates for the latter four parameters both within the particle filter, and by MCMC (*Table 1B legend*).

### Implementation and distribution

We include details of the implementation of the EpiFusion algorithms in S2 Text, including pseudocode for the MCMC and particle filtering algorithms. The EpiFusion model is distributed as a Java program and the model source code, executable files, tutorials, example parameter files and guidance on usage are available at the GitHub repository, https://github.com/ciarajudge/EpiFusion, under the GNU General Public License. The program takes an XML file as input, which contains the data and parameters for the model. The user does not need to define any compartmental model (i.e. SIR, SEIR etc), but parameterisation of rates *β*,*γ*,*φ* and *ψ* is necessary with a selection of options available to users for priors or to allow discrete step-changes in these rates at specific times during the outbreak (e.g., corresponding to known dates of changes in public health surveillance strategies). Code used for the models and plots in this manuscript are housed at the GitHub repository https://github.com/ciarajudge/EpiFusion_PublicationRepo.

### Model validation and testing

We validated and tested the performance of EpiFusion using five different approaches:: *(i)* comparison of the EpiFusion phylodynamic likelihood to the BEAST2 BDSky phylodynamic likelihood to validate our novel likelihood calculation *(ii)* large scale (i.e., many replicates) simulation based calibration [33,45], *(iii)* scenario testing, *(iv)* noise testing, and *(v)* benchmarking of accuracy against existing models.

#### Simulated datasets

**Fig 2:**
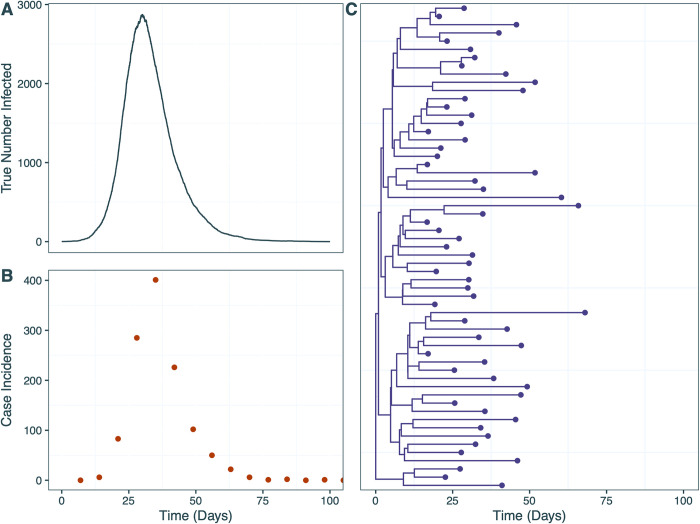
Example ReMASTER epidemic simulation and resulting data used for the EpiFusion program (specifically, according to the “Baseline Scenario” described in the section “Scenario Testing”). (a) True number of people infectedover time, from which (b) weekly reported case incidence counts and a (c) phylogenetic tree of simulated samples were derived based on given sampling rates. Plots of the other simulated datasets are provided in S2–S4 Figs.

The latter four phases involved the use of simulated epidemic datasets with SIR transmission dynamics that were generated using ReMASTER [46]. ReMASTER produces the true trajectories over time of each population compartment (S, I and R), identified cases over time (which we aggregated into weekly case incidence), and a phylogenetic tree of all identified cases under the epidemiological sampling rate, which we downsampled to give a simulated phylogeny of sequenced samples with a smaller sampling rate (*Fig 2*). Details of the simulated datasets used in this manuscript are provided in the Supplementary Information (*S1 and S2 Tables* and *S2–S4 Figs*).

#### Likelihood comparison

To validate our daily approximation of the phylodynamic likelihood we compared the EpiFusion likelihoods to those computed with the BEAST2 [47] package BDSky (13). We examined the effect on the likelihood of varying the parameters *β*,*γ*, and *ψ* in turn around their true values with all other parameter values fixed to the truth. We repeated this on a range of simulated datasets with varying true values of each parameter. To evaluate the estimation of the infection or birth rate parameter (*β*), we used datasets generated under a constant-rate birth-death process in ReMASTER [46].

#### Simulation based calibration

To assess calibration of our MCMC algorithm, we defined distributions of the EpiFusion model parameters *β*,*γ*,*φ* and *ψ* and simulated 500 unique epidemics using parameter combinations drawn randomly from these distributions. We then fit EpiFusion models with priors equal to the original distributions from which the parameters were drawn, and analysed the ability of EpiFusion to recapture the true parameter values within Highest Posterior Density (HPD) intervals of increasing credible mass. A perfectly calibrated MCMC algorithm should result in 5% of models capturing the true parameter in their 0.05 HPD intervals, 10% of models capturing the true parameter in their 0.10 HPD intervals, etc. The *β* parameter varies over time in both our model and the simulated data (i.e. it is modelled as *β*_*t*_), as opposed to consisting of one fixed value per simulation. Thus, to calculate coverage at a given value of credible mass alpha for the *β* parameter, we took the average proportion of the true *β*_*t*_ trajectory that falls within the inferred HPD interval across all replicates.

#### Scenario testing

We examined the ability of EpiFusion to reconstruct infection and R_t_ trajectories under a range of epidemic scenarios. The parameters under which each of these scenario datasets were simulated are included in the Supplementary Information (*S2 Table*). To assess the advantage of combining phylodynamic and epidemiological data in this framework, models using solely the phylogenetic tree or case incidence data were compared to using a combination of both (*S3 and S4 Tables*). The three scenarios examined were: *(i)* the introduction of a novel pathogen into an immune naïve population with time-constant sampling, *(ii)* an introduction scenario with a step-change in sampling when the outbreak is ‘discovered’, and *(iii)* a step-change in transmission of an endemic pathogen that has previously circulated at stable levels. We assessed model performance according to a selection of metrics and probabilistic scoring rules (*Table 2*). Further details on the performance metrics used and how they were calculated are included in the Supplementary Information (*S5 Table*).

**Table 2:** Statistics used to evaluate model performance under scenarios 1, 2, and 3 for analyses using case incidence only (epi), phylogenetic tree only (phylo), and both data sources combined (combo). The best or joint-best result for each statistic for each scenario is highlighted in bold. Trajectory RMSE: root-mean-squared error. Calibrated Trajectory Coverage: proportion of true trajectory that falls within the 95% HPD, scaled by 0.95. Scaled HPD Width: mean width of the 95% highest posterior density interval, scaled by the true value. Continuous Ranked Probability Score: mean CRPS across the trajectory time series. Brier Score: Classification accuracy for transmission phase (Rt) being above or below 1. Further details on the calculation of these statistics are included in the Supplementary Information (S5 Table).

	*Baseline Scenario*			*Sampling Step-Change*			*Transmission Step-Change*		
Evaluation Metric	*Epi*	*Phylo*	*Combo*	*Epi*	*Phylo*	*Combo*	*Epi*	*Phylo*	*Combo*
Infection Trajectory RMSE	43.2	329.8	**41.3**	372.7	**137.1**	185.3	196.07	171.19	**131.41**
Infection Trajectory Coverage	1.05	1.05	**1.04**	0.93	0.97	**1**	**1.01**	0.95	1.05
Scaled Infection Trajectory HPD Width	1.94	1.41	**1.13**	1.07	1.26	**0.96**	1.03	1.24	**0.84**
Rt Trajectory RMSE	0.333	0.356	**0.217**	0.299	0.343	**0.222**	0.291	0.349	**0.266**
Rt Trajectory Coverage	**1.02**	1.05	1.05	**1.05**	**1.05**	**1.05**	**1.03**	1.04	1.04
Scaled Rt Trajectory HPD Width	**1.52**	1.55	1.66	**1.46**	1.55	1.66	**1.16**	1.56	1.49
Continuous Ranked Probability Score	88.19	162.91	**41.04**	265.92	**158.68**	182.95	109.25	114.23	**83.44**
Rt Continuous Ranked Probability Score	0.188	0.196	**0.129**	0.181	0.183	**0.123**	0.158	0.199	**0.15**
Brier Score–Transmission Phase	0.034	0.042	**0.011**	0.031	0.032	**0.019**	0.105	**0.101**	0.109

#### Noise testing

We then tested model robustness to noise, by testing scenarios with increasing transmission or observation noise and examining the effect on the inferred R_t_ continuous ranked probability score. Here we use the term transmission noise to mean fluctuations in R_t_, and observation noise to mean fluctuations in the case and sequence sampling rate. We achieved increasing noise in the ReMASTER simulations by replacing constant transmission or sampling rates with a time series of rates drawn from Gaussian distributions with increasing standard deviations (*S2 Table*).

#### Benchmarking against existing approaches

For the three scenarios outlined in ‘Scenario Testing’, we benchmarked the combined EpiFusion model against existing packages EpiNow2 [48], BDSky [13] and TimTam [29,40] which are respectively among the most commonly used tools for estimating R(t) from epidemiological, phylodynamic, and both data types. The BDSky and TimTam models are usually provided with a sequence alignment as input data and subsequently infer phylogenetic trees. Here, we instead directly provided these models with the same fixed tree as was provided to EpiFusion (i.e., a phylogeny down-sampled from the tips in the simulated true transmission tree). This removed phylogenetic uncertainty to allow a fairer comparison of the model performances. Full model specifications are in the Supplementary Information (*S3 Text*). As the BDSky and TimTam models require specification of intervals in which to infer R(t), uniform intervals of 5 or 10 days were provided. It was necessary to use different specifications of R_t_ intervals for the TimTam and BDSky approaches across different scenarios due to a particular sensitivity in TimTam to the interval change times, where placing the intervals at certain points resulted in highly impractical estimates.

### Ebola virus disease in Sierra Leone

Finally we used an EpiFusion combined model with a negative binomial epidemiological observation model to infer R_t_ over the course of the 2014 Ebola virus outbreak in Sierra Leone. We obtained case count data from Fang et. al [49] and a maximum clade credibility tree generated from a BEAST Coalescent Skygrid analysis with an uncorrelated lognormal relaxed clock from the Github repository associated with Dellicour et. al [50]. The tree contained samples from Sierra Leone, Guinea and Liberia, so we selected a monophyletic clade of 980 Sierra Leone sequences (*S5 Fig*). We aggregated the case count data (total 8358 confirmed cases) into weekly incidence to reduce any observation noise introduced by weekly periodicity in reporting rates (*S6 Fig*), and used a combined EpiFusion model to estimate national R_t_ from March 2014 to August 2015 (78 weeks). We fit *β* as a series of linear splines (see Table 1B legend), and *γ* as a constant value over time. The model was run using 6 chains of 10,000 MCMC samples with 300 particles each.

## Results

### Testing on simulated data

#### Likelihood comparison

**Fig 3:**
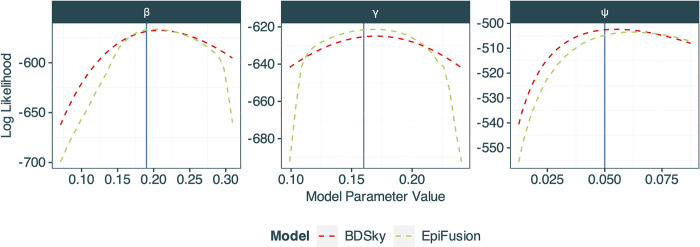
Comparison of median log likelihoods generated by EpiFusion (green) and a birth-death skyline model implemented in the BEAST2 (50) package BDSky (14) for the parameters *β*,*γ* and *ψ*. The true value of the parameter is marked by the blue vertical line.

Our comparison of the phylodynamic likelihood calculated by EpiFusion with that calculated in BDSky shows good agreement between the two approaches *(Fig 3)*, though the stochastic and approximate nature of the EpiFusion likelihood means that the values are not identical. The EpiFusion likelihood curves are also less smooth due to the stochastic nature of the algorithm. As the parameter values get further from the truth for the *β* and *γ* parameters, the EpiFusion likelihood drops sharply due to the parameter values implying highly unlikely or even impossible trajectories. More extensive likelihood comparisons are available in the Supplementary information *(S7 Fig).*

#### Simulation based calibration

**Fig 4:**
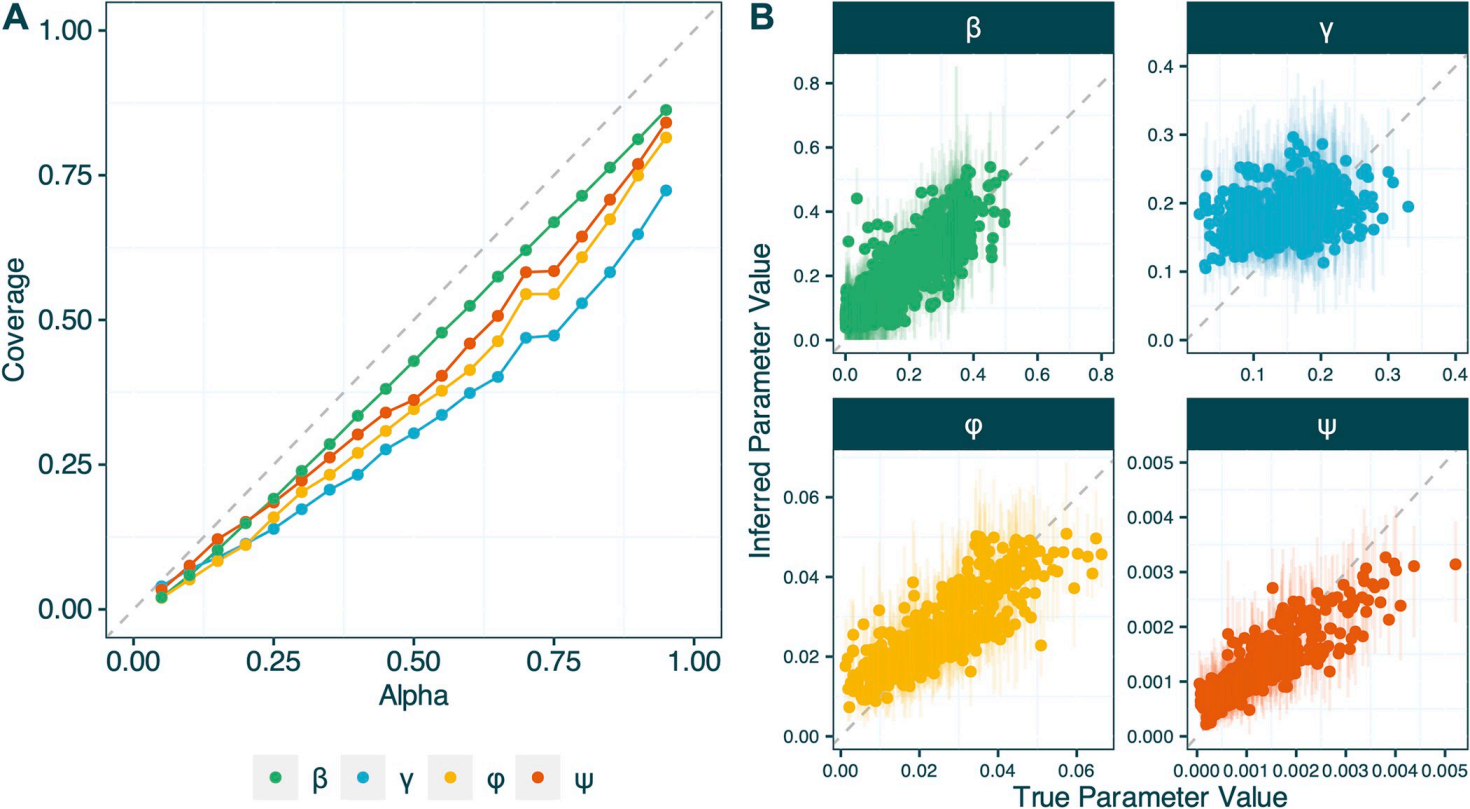
(a) Proportion of replicates that capture the true value of the parameter within their HPD intervals (y-axis) of increasing credible mass alpha (x-axis), for the parameters: *β* infection parameter (green), *γ* recovery parameter (blue), *φ* case sampling rate (yellow) and *ψ* sequence sampling rate (orange). For the infection rate parameter *β* (*which varies over time*), the y-axis reflects the average proportion of the *β* trajectory captured in the HPD interval across all replicates (b) Mean inferred value and 95% HPD interval of the parameter (y-axis) plotted against the true value of the parameter (x-axis). For the infection parameter *β*, a subset of 1000 values of *β*_*t*_ is shown for clarity in the plot as *β* varied over time in the simulations and models, so each replicate resulted in the inference of many *β*_*t*_ values. For both graphs the grey dotted line indicates the ‘perfect’ result: perfect calibration for (a) and perfect agreement between true and inferred parameters for (b).

In Fig 4 we show the results of the simulation-based calibration of the combined EpiFusion model. Fig 4A shows the proportion of replicates (or ‘coverage’) that recover the true parameter with increasing credible interval mass (‘alpha’). We note that coverage increases with increasing credible interval mass, however slight under-coverage is observed, particularly for the *γ* parameter. This is also demonstrated in Fig 4B, where the model appears to have limited ability to estimate the *γ* parameter. However, the model does appear to recapture the true values of the sampling parameters *φ* and *ψ*, with only slight underestimation for larger true values. The model was generally able to accurately infer the values of *β* over time.

#### Scenario testing

Next we evaluated how well EpiFusion could reconstruct trajectories of infections and R_t_ corresponding to simulated outbreaks reflecting three common epidemiological scenarios: *(i)* the introduction of a novel pathogen into an immune naïve population with time-constant sampling, *(ii)* an introduction scenario with a step-change in sampling when the outbreak is ‘discovered’, and *(iii)* a step-change in transmission of an endemic pathogen. We compared the performance of EpiFusion using as input solely case incidence data, solely a phylogenetic tree, and using both datasets combined. The metrics by which models are compared and their statistics are summarised for a single realisation of each scenario in Table 2.

**Fig 5:**
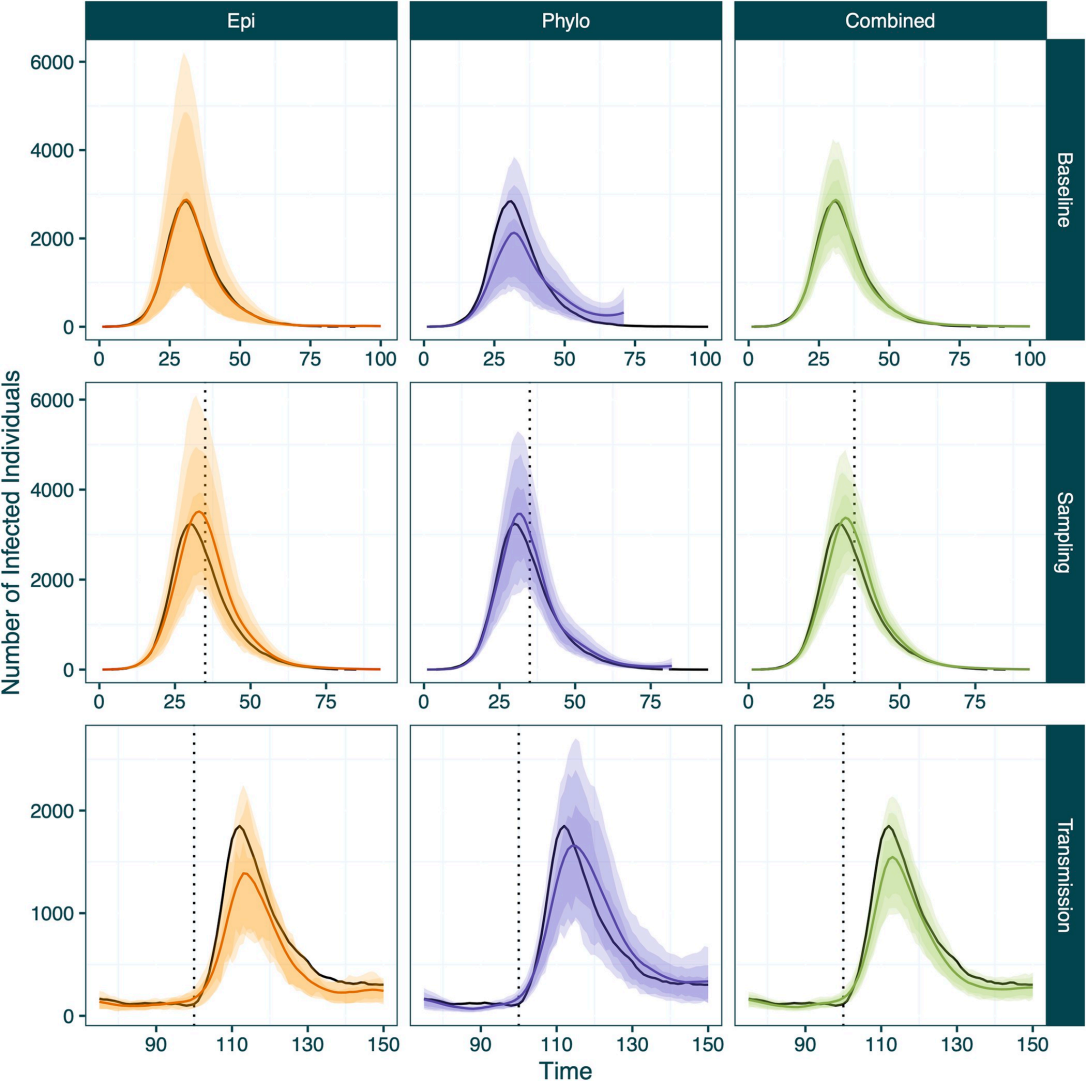
Inferred mean infection count trajectories from EpiFusion using only case incidence (orange), only the phylogenetic tree (purple) and both data types combined (green) (columns) for the three scenarios tested (rows). The true number infected over time is represented by the black line. 95%, 80% and 66% highest posterior density intervals are represented by increasingly dark shaded regions. Times of step-changes are marked by the vertical dotted lines for the step-change in sampling and transmission scenarios: a 10-fold increase in case and genomic sequence sampling rates on day 35 for the ‘Sampling’ step-change scenario, and a 3-fold increase in transmission rate on day 100 for the ‘Transmission’ step-change scenario.

**Fig 6:**
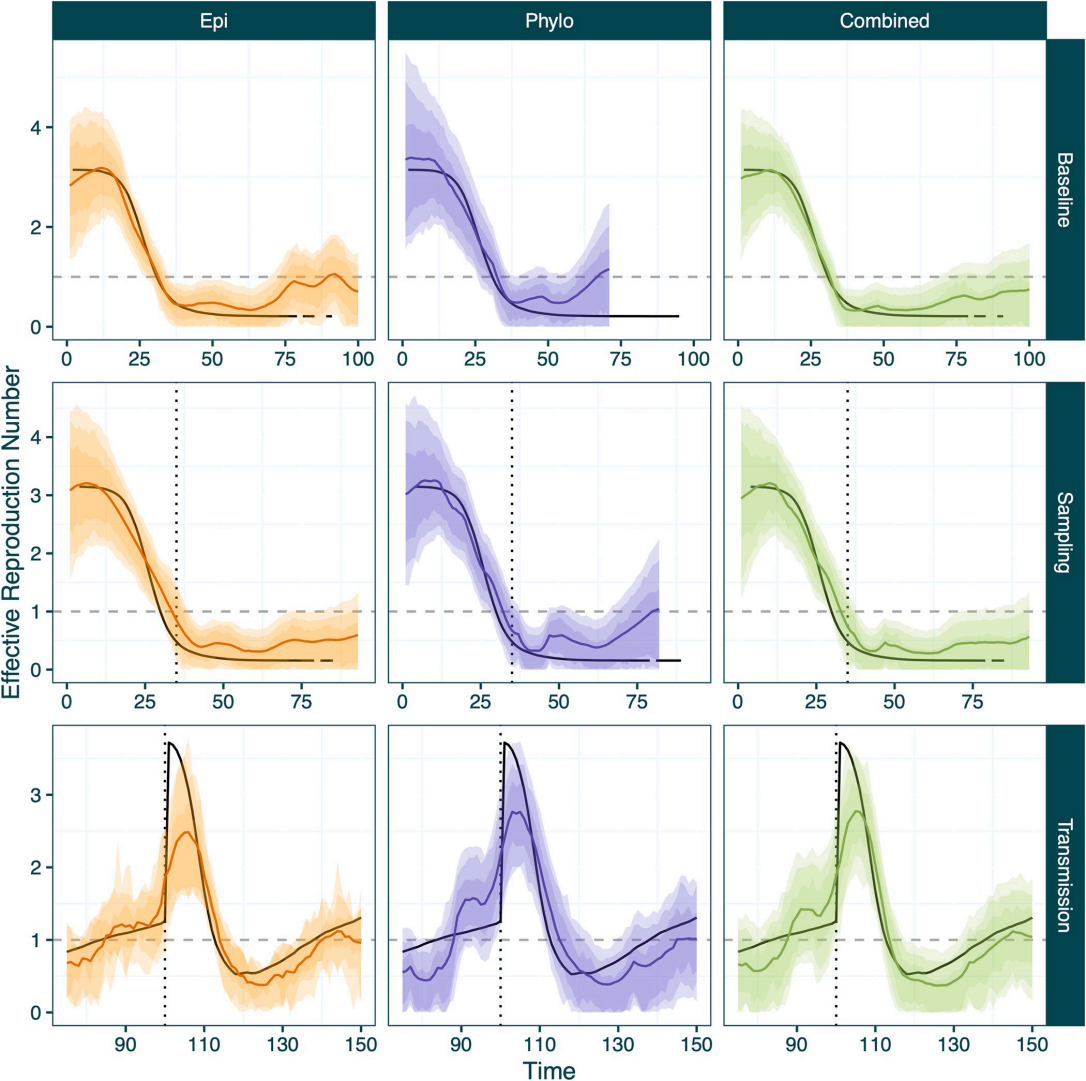
Inferred R_t_ from EpiFusion using only case incidence (orange), only the phylogenetic tree (purple) and both data types combined (green) for the three scenarios tested (rows). True R_t_ is represented by the solid black line. 95%, 80% and 66% highest posterior density intervals are represented by increasingly dark shaded regions. Times of step-changes are marked by vertical dotted lines: a 10-fold increase in case and genomic sequence sampling rates on day 35 for the ‘Sampling’ step-change scenario, and a 3-fold increase in transmission rate on day 100 for the ‘Transmission’ step-change scenario. An R_t_ of 1 is marked by the dashed horizontal line. The true R_t_ fluctuates at the end of the sampling step-change scenario due to very low prevalence as the outbreak ends.

We first considered a scenario in which a novel pathogen enters an immune naïve population with constant sampling: the ‘baseline scenario’. Here, each approach successfully captured the true epidemic and R_t_ trajectories within the 95% HPD intervals (*Figs 5 and 6*), however the tree only approach underperformed compared to the case incidence only and combined approaches according to the metrics that we chose for evaluation (*Table 2*). The combined approach was most successful in estimating the true infection trajectory (Infection Trajectory RMSE: *41*.*3*) compared to tree only and case incidence only models (*329*.*8*, *43*.*2*) (see Tables 2 and S5 for a description of the statistics). These improvements in infection trajectory estimation are accompanied by a reduction in the width of the scaled HPD intervals (*1*.*13* vs *1*.*41* and *1*.*94*), a positive result indicating increased confidence, provided that coverage and accuracy is maintained (as is observed here). The Continuous Ranked Probability Score (CRPS) was used to evaluate the probability of the true infection or R_t_ trajectory given the posterior infection or R_t_ trajectories from each model, where a lower value equates to a more accurate result. Here the combined approach also performed best for both infection and R_t_ trajectories (*41*.*04* vs *88*.*19* and *162*.*91* for infection trajectories and *0*.*129* vs *0*.*188* and *0*.*196* for R_t_ trajectories).

Each of the approaches demonstrated a slight propensity to over-cover the infection and R_t_ trajectories (calibrated trajectory coverages > 1). The combined approach led to a decrease in R_t_ trajectory RMSE (*0*.*217* vs *0*.*333* and *0*.*356*). We also used the Brier score (mean squared error between the probabilistic prediction and the true outcome) to evaluate each approach based on its ability to predict transmission phase, i.e. correctly estimating if R_t_ is above or below 1, where a lower Brier score indicates improved performance. We find each approach to be adept at classifying R_t_ as above or below 1, however the combined approach (*0*.*011*) leads to a marked improvement compared to the case incidence only or tree only approaches (*0*.*034*, *0*.*042*).

The second scenario consisted of a simulated outbreak with similar transmission dynamics to the introduction scenario but for which levels of both genomic and case sampling are low during the initial period of spread until more widespread surveillance is introduced (thus leading to a step-wise increase in sampling density). This was characterised in the data simulation by a spontaneous 10-fold increase of the case and genomic sequence sampling rates on day 35 of the simulation (*S2 Table*). Here, the date of this step-change is provided as a fixed parameter to the model under the assumption that it would be known to health authorities, but fixing this parameter is not strictly necessary to run the model as it can be co-inferred with MCMC by providing the model with an expected number of step-changes in sampling rates. The sampling rates before and after the step-change are inferred as parameters of the MCMC.

For this analysis, all three approaches successfully infer the R_t_ trajectories (*Fig 6*), but slightly overestimate the peak of the infection trajectories, with the case incidence only approach being the least accurate. This is further reflected in the performance metrics (*Table 2*), where the case incidence only approach performs the best for only one metric, scaled R_t_ trajectory HPD width. The combined approach demonstrates optimal scaled coverage of the true infection trajectory (*1*), while at the same time reducing the HPD interval width (*0*.*96 vs 1*.*07*, *1*.*26*) in comparison to individual approaches (*Fig 5*). The combined approach also led to the best R_t_ trajectory CRPS results (*0*.*123* vs *0*.*181* and *0*.*183*) by a wide margin and led to a reduction of almost 50% in the Brier score (*0*.*019* vs *0*.*031* and *0*.*032)*. The tree only approach demonstrated more advantages in this scenario than in the other scenarios, resulting in the best infection trajectory RMSE and CRPS (*137*.*09* and *159*.*68*, respectively).

The final scenario examined was a scenario in which a step-change in transmission was simulated, such as when a pathogen experiencing endemic transmission undergoes a rapid increase in transmission, but where sampling parameters remain constant. Specifically, we simulated an outbreak scenario where the transmission rate was increased 3-fold on day 100 of the simulation *(S2 Table).* For this analysis, the date of the transmission increase was inferred as a parameter of the MCMC (it is possible to fit any number of rate step-changes with EpiFusion; it is not currently possible to infer the number of step changes). All three analyses broadly captured the epidemic trajectories (*Fig 5*), with the case incidence only approach demonstrating better coverage (*1*.*01*, vs *0*.*95*, *1*.*05*), however the combined approach resulted in the lowest trajectory RMSE (*131*.*41* vs *196*.*07* and *171*.*19*) and CRPS (*83*.*44* vs *109*.*25* and *114*.*23*). The combined approach also resulted in a slightly improved CRPS (*0*.*15* vs *0*.*16* and *0*.*20*), along with improved R_t_ RMSE (*0*.*266*, vs *0*.*291* and *0*.*349*). The Brier score for this scenario is the only instance across all metrics and scenarios where the combined approach did not result in an improvement or perform equally to one or both individual approaches. However, the difference between all three approaches for this metric is marginal (*0*.*105*, *0*.*101*, *0*.*109* for case incidence only, tree only and combined approaches, respectively).

#### Noise testing

**Fig 7:**
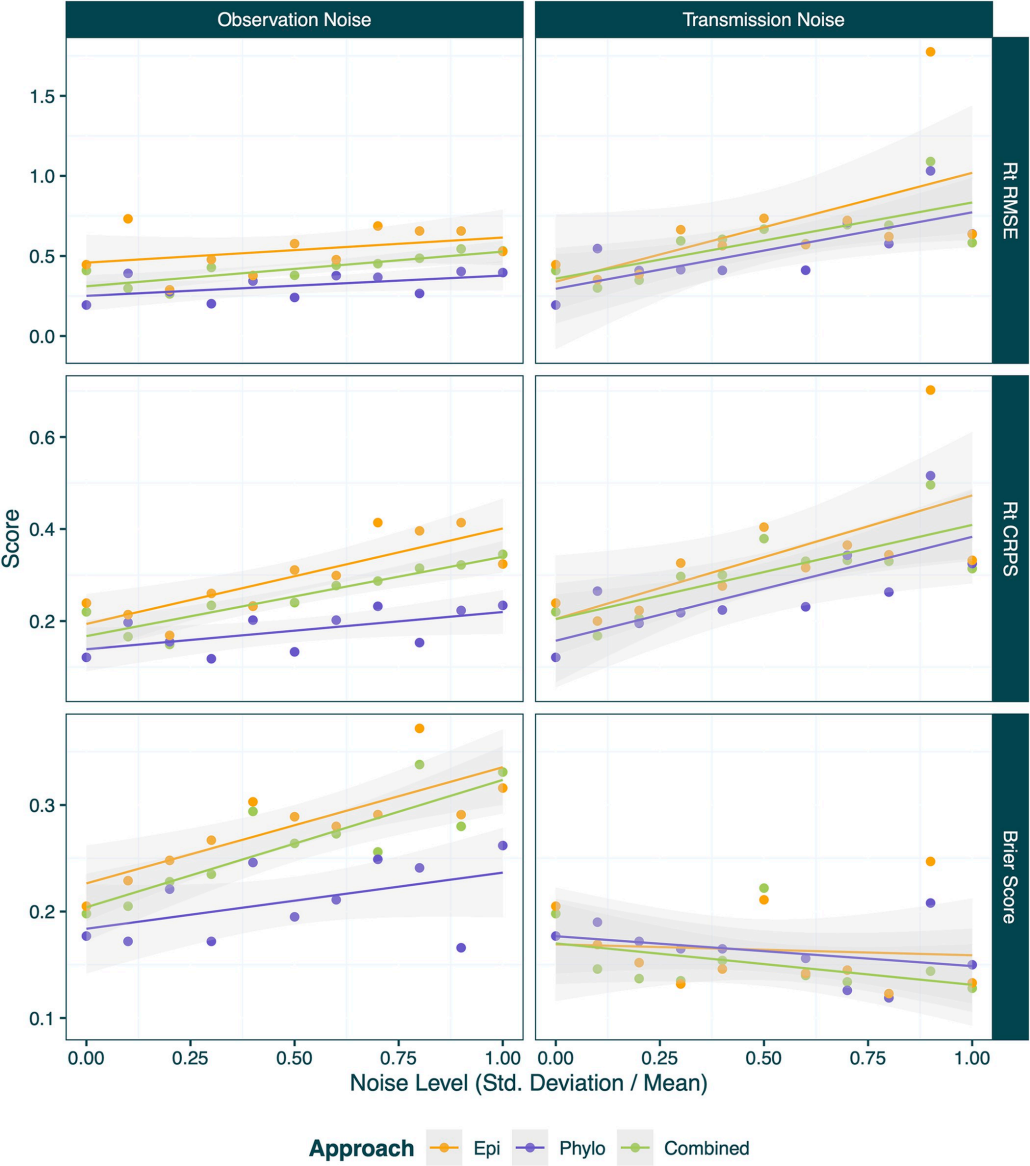
R_t_ trajectory RMSE, CRPS and Brier Score (y-axes) for case incidence only (orange), tree only (purple) and combined (green) EpiFusion approaches on scenarios with increasing noise (x-axes). For each of these metrics, a value closer to 0 reflects a better score. Noise is quantified as the standard deviation divided by the mean of the distribution from which the transmission or sampling rates were drawn. The general trend is shown by linear regression lines of the corresponding colour.

Next we examined the performance of the three approaches on scenarios with increasing observation and transmission noise, and summarise the results by examining how the R_t_ RMSE, CRPS, and Brier Score changes (*Fig 7*). R_t_ trajectory fits for these scenarios are included in the Supplementary Information (*S8 and S9 Figs*). The tree only approach appears most robust to observation noise. Each metric sees a decrease in performance with increasing noise, with the exception of the Brier Score, which improves with increasing transmission noise.

#### Benchmarking against existing approaches

**Fig 8:**
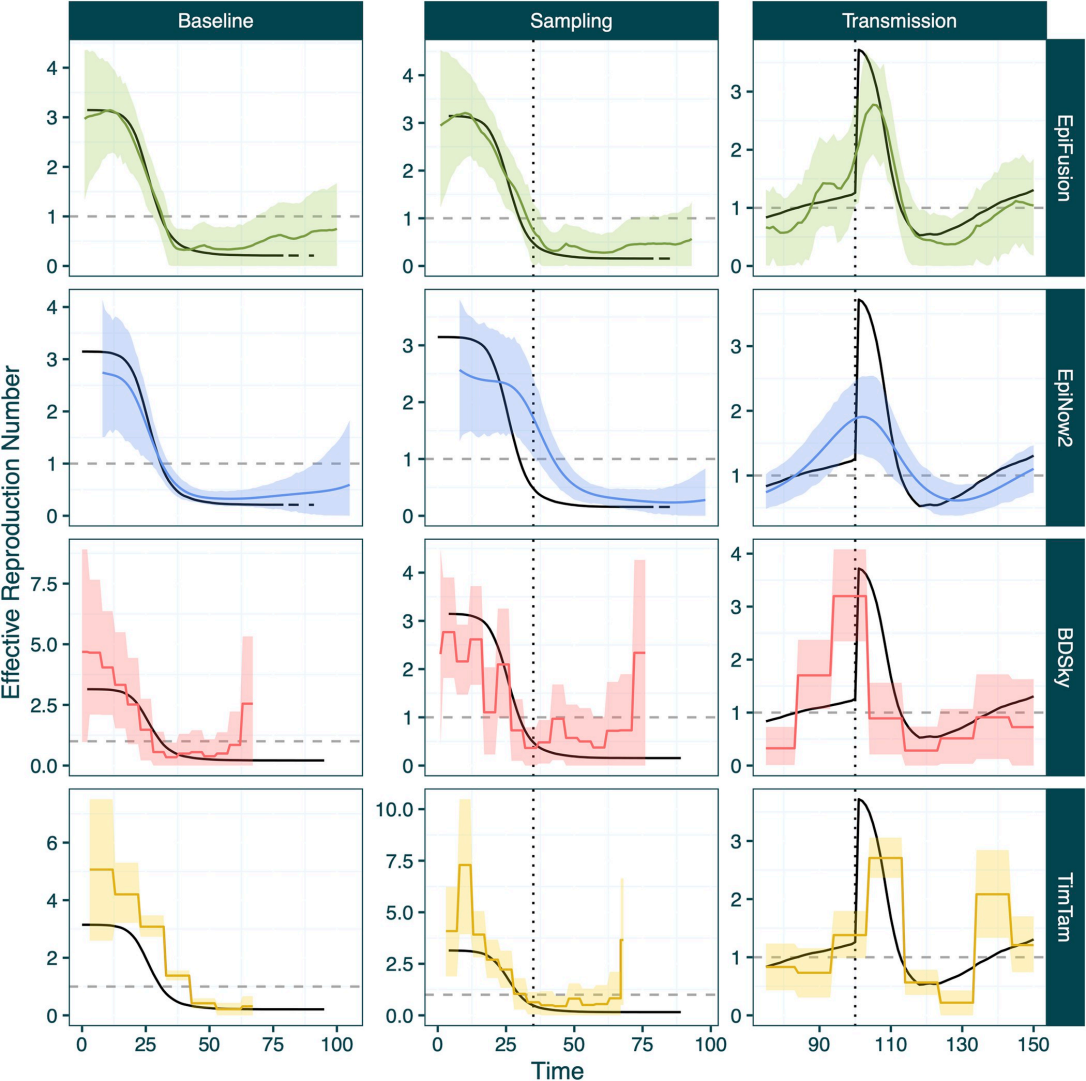
Estimated mean Rt and 95% HPD intervals for the three validation scenarios from EpiFusion (green), EpiNow2 (blue), BDSky (red) and TimTam (yellow).

We compared the performance of the EpiFusion combined model against existing R_t_ inference methods (*Fig 8*) on the simulated datasets from the scenario testing section. We used (i) EpiNow2 [48], (ii) a Birth-Death Skyline Serially Sampled model implemented in BEAST2 (BDSky) [13], and (iii) TimTam [29,40] implemented in BEAST2 to represent commonly used approaches for estimating R(t) from only molecular data, case incidence data, and both data types. Further information on model specifications is included in the Supplementary Information (*S3 Text).*

R_t_ posteriors were obtained from each pre-existing tool for all three scenarios and compared to the combined EpiFusion approach. The strengths and weaknesses of the different models are apparent when examining their performance under selected scoring criteria (*Table 3*).

**Table 3:** Model Benchmarking.

	*Introduction Scenario*				*Sampling Step-Change*				*Transmission Step-Change*			
	*EpiFusion*	*EpiNow2*	*BDSky*	*TimTam*	*EpiFusion*	*EpiNow2*	*BDSky*	*TimTam*	*EpiFusion*	*EpiNow2*	*BDSky*	*TimTam*
Rt RMSE	**0.22**	0.22	0.91	1.17	**0.22**	0.58	0.86	1.38	**0.37**	0.54	0.92	0.74
Rt CRPS	0.13	**0.11**	0.4	0.72	**0.12**	0.28	0.45	0.542	**0.15**	0.42	0.45	0.76
Brier Score	**0.011**	0.145	0.178	0.226	**0.109**	0.236	0.237	0.227	0.109	0.417	**0.103**	0.143
Rt Coverage	1.05	1.05	**1.04**	0.57	**1.05**	0.55	0.89	0.94	**1.02**	0.82	0.66	0.61

Each model captured the general trend of transmission for all three scenarios, with some weaknesses. Using EpiFusion resulted in improved R_t_ RMSE for all three scenarios. EpiFusion also led to substantially improved Brier scores compared to other methods for the introduction and sampling scenarios. For the sampling and transmission scenarios, EpiFusion resulted in improved R_t_ CRPS by a large margin, and the best coverage by a smaller margin. Notably EpiFusion never produced the worst performance under any scenario and metric combination. EpiNow2 performed well in the introduction scenario, yielding the best R_t_ CRPS, however the model somewhat struggled with identifying the sharp fluctuations in transmission in the third scenario, especially the initial step-change in transmission, possibly due to the smoothing influence of the Gaussian process. For the sampling scenario it was not possible to parameterise the large and sudden step-change in sampling in the EpiNow2 model. This is reflected by the underperformance of EpiNow2 in this scenario, where the sharp increase in case incidence due to increased sampling is instead interpreted by the model as sustained transmission of R_t_ > 1 (*Fig 8*). The BDSky approach systematically overestimated R_t_ towards the end of the time series, a problem which interestingly also affected the EpiFusion tree only model fits (*Fig 6*). However, the model generally demonstrated good coverage of the true R_t_, despite inferring the parameter in piecewise constant intervals. Conversely, TimTam struggled with slight overestimation of R_t_ at the beginning of the time series.

### Ebola virus disease in Sierra Leone

**Fig 9:**
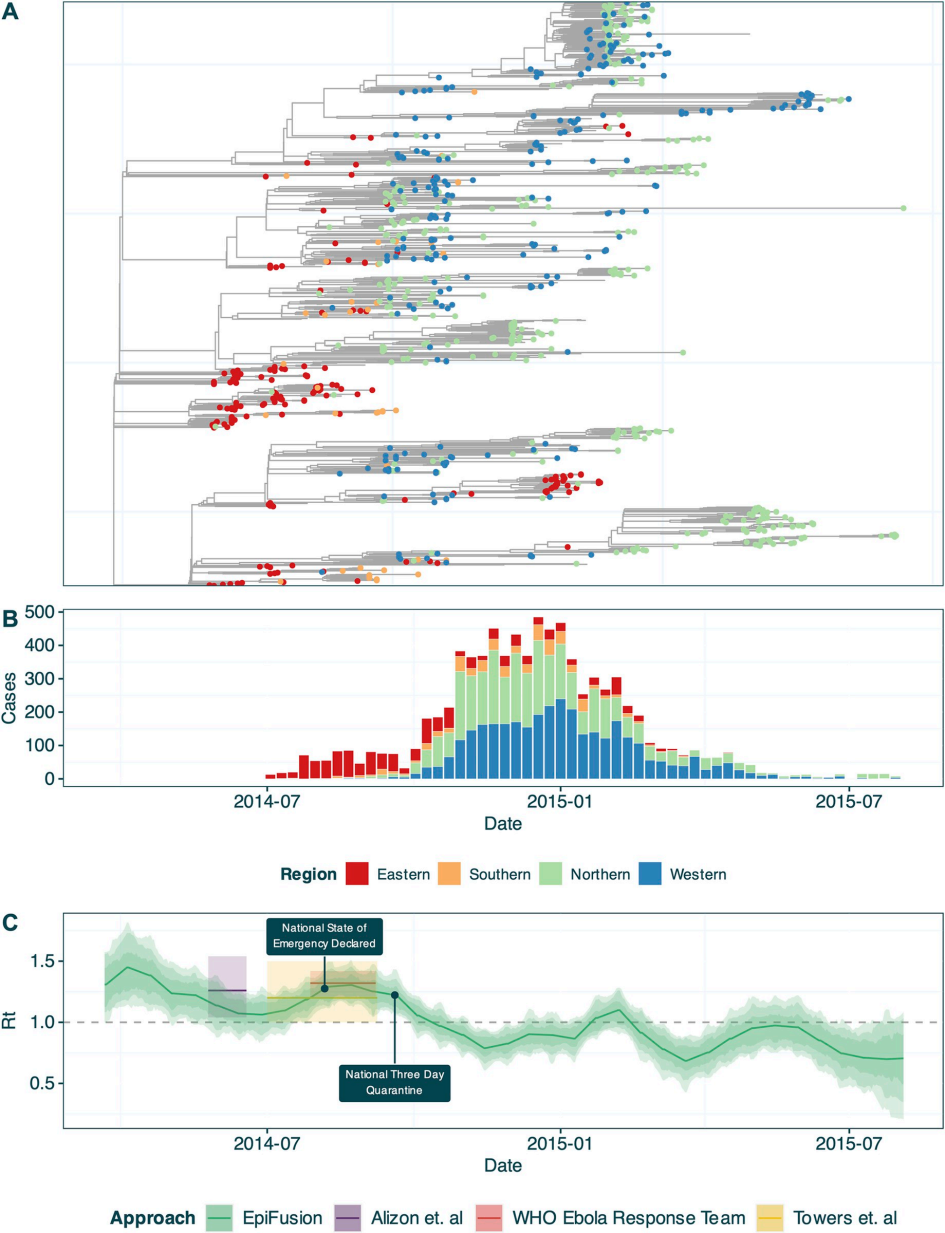
(a) Phylogenetic tree of Ebola virus sequences in Sierra Leone consisting of a subclade of the MCC tree obtained from Dellicour et. al [50], with tips coloured by region at a 1^st^ administrative unit level. (b) Weekly case incidence of Ebola virus disease in Sierra Leone obtained from Fang et. al [49], stratified by region. (c) Inferred median effective reproduction number (solid line) of Ebola virus disease in Sierra Leone from an EpiFusion combined model. 95%, 80% and 66% highest posterior density intervals are represented by increasingly dark shaded regions. Two key dates in the epidemic are labelled: (i) Declaration of a national state of emergency on August 6^th^ 2014, and (ii) national three day quarantine beginning on September 19^th^ 2014.

Finally, we demonstrated the use of an EpiFusion combined model on real data by retrospectively inferring the R_t_ of Ebola virus in Sierra Leone from March 2014 to August 2015 (*Fig 9*). The root of the tree was in March 2014, approximately two months prior to the first observed epidemiological case, allowing us to model the early dynamics of the outbreak. The EpiFusion analysis was completed within 9 hours on a MacBook Air M3 PC with an 8 core CPU. We expect that the long duration of the time series (>1.5 years) influenced the runtime.

We estimate the initial R_t_ during the first week of the study time series to be 1.33 (with lower and upper 0.95 HPDs of 1.04 and 1.61 respectively). Fig 9C shows that the R_t_ trajectory inferred by EpiFusion is in agreement with other estimates in the literature [47,51–53] including a birth-death phylodynamic approach implemented by Alizon et. al [52], and epidemiological models used by Towers et. al [51] and the WHO Ebola Response Team [53]. The average daily reproductive number by Wiratsudakul et. al [54] for the first year of the outbreak was comparable to our estimate over the same time period (1.03 vs 1.08), with the estimates in this paper also mirroring the small uptick in R_t_ we observe in early 2015. However, EpiFusion infers a slightly later time period for the decrease of R_t_ below 1.0 (13^th^ October, 0.95 HPD 18^th^ September– 5^th^ November) than some other studies (Althaus et. al ‘late July’ [55], Nishiura et. al, ‘late August’ [56]).

The trajectory also aligns well with key dates [49] during the outbreak, particularly the three day nationwide quarantine on September 19^th^ 2014 [57] which is followed by a sharp drop in the inferred R_t_ of our model.

## Discussion

We outline EpiFusion, a computationally tractable and flexible infrastructure for the combination of phylogenetic and epidemiological data to estimate infection and R_t_ trajectories. EpiFusion fills a gap in current modelling approaches at the intersection between the fields of phylodynamics and epidemiology (*Table 4*). We show that by combining data types with EpiFusion it is often possible to improve the accuracy of R_t_ or infection trajectory estimates compared to using only phylogenetic or epidemiological data alone.

**Table 4:** Comparison of the key characteristics of EpiFusion compared to the tools and literature referred to in this manuscript. Rasmussen’11 denotes Rasmussen et. al (2011), which was referenced in the introduction. However, the model is not distributed for use as a software or program, so we were unable to assess its computational efficiency (*). (BD–birth death).

Qualitative Comparison to Other Tools or Models						
	*Uses Case Incidence*	*Uses Phylogenetic Tree*	*Infers Phylogenetic Trees*	*Infers Continuous Trajectories*	*Scalable to Large Datasets*	*BD Theory*
EpiFusion	✔	✔		✔	✔	✔
EpiNow2	✔			✔	✔	
EpiInf	✔	✔	✔	✔		✔
TimTam	✔	✔	✔		✔	✔
BDSky		✔	✔		✔	✔
Rasmussen ‘11	✔	✔		✔	*	

Through extensive simulations we found the EpiFusion model to be adept at recapturing the case incidence and genomic sampling parameters *φ* and *ψ*. The model was less able to accurately recapture the *γ* recovery parameter, but this can often reliably be obtained from empirical literature [57,58], and thus could be informed in practice with a strong prior. Given that the EpiFusion process model simulates epidemic trajectories according to the balance of the infection and recovery parameters *β* and *γ*, we suspect the flexible specification of time-varying *β* disincentivises accurate inference of the *γ* parameter. However, while we would expect *β* to also be biased in the opposite direction to *γ* under this hypothesis, Fig 4B indicates that the model is capable of accurately inferring the true value of *β* over time and the time-varying nature of *β* in the model and simulated data made it difficult to fully characterise any bias in the parameter. Nevertheless, although the model does not consistently recover the *γ* parameter, it does reliably reconstruct infection and R_t_ trajectories over time *(Figs 5, 6 and S10).* Future development of EpiFusion will aim to improve coverage of epidemiological parameters.

When testing the ability of EpiFusion to recover changes in R_t_ in different epidemiological scenarios (Scenario Testing section) the ‘baseline scenario’ aimed to represent a situation such as the emergence of a novel pathogen [59] or the expansion of an existing pathogen into a new ecological niche [60]. All three EpiFusion approaches (case incidence only, tree only, combined) were able to accurately reconstruct the epidemic trajectories of the simple, single epidemic peak, with the combined approach resulting in the best result for seven of the nine performance metrics tested. While the outbreak lasted 100 days, the inference using the phylogenetic tree is truncated at day 69 of the simulation as this is the date of the last sampling event on the tree. An advantage of the combined model is therefore that the trajectories can be jointly inferred up until the last sampling event on the tree, but after this point R(t) can still be estimated using any additional case incidence data only (as is often the case in real-time outbreak response, where recent case incidence data usually precedes new genomic sequences). Conversely, where the most recent common ancestor of viruses sampled is phylogenetically estimated prior to the first observed case (as in the Ebola example we show here (*Fig 9*), it is possible to infer R(t) for earlier time points than possible for case incidence only approaches.

We subsequently considered more complex scenarios in which the sampling or transmission rates change over time in a more realistic way. Such changes are widely acknowledged to complicate the estimation of R_t_. This allowed us to examine how combining phylodynamic and epidemiological models and data could improve our ability to accurately estimate R_t_ under such challenging scenarios. The rationale for the step-change in sampling scenario was to emulate the transition of a disease from passive to active surveillance, perhaps due to the declaration of a Public Health Emergency of International Concern (PHEIC), resulting in a lack of data from the early stages of an outbreak and a lack of comparability in case numbers before and after detection is scaled up. This also applies for novel pathogens that do not have established means of clinical diagnoses or reporting, or where testing is initially limited. For example, during the Zika virus epidemic in Brazil in 2016, case detection rates rose sharply following the implementation of widespread PCR testing [61], compared to the beginning of the outbreak. The tree only approach demonstrated more advantages during this scenario than in the other scenarios tested, which is likely due to the additional information captured by birth events in the tree even when sampling was low. Notably the combined approach led to improved R_t_ continuous ranked probability scores, the probabilistic scoring rule we chose for model comparison. For both the baseline and sampling scenario the combined model greatly outperformed the individual approaches according to the Brier score metric, leading to ~4 fold and ~2 fold decreases in the baseline and sampling scenarios, respectively. This indicates that the combined approach may benefit estimation of whether an epidemic is growing or declining, which is a useful public health indicator to be able to evaluate with certainty [62,63].

The step change in transmission scenario was used to mimic a sudden increase in transmission, such as a change in human behaviour (e.g. school holidays end, non-pharmaceutical intervention ceases), or a change in the intrinsic transmissibility of a pathogen (e.g. a new variant [64]). The phylogenetic tree simulated from ReMASTER is more applicable to the former, in that all ‘active’ lineages at the time of the step-change undergo an equal increase in transmission which is not what would be observed in the case of a new, more transmissible variant. Currently, EpiFusion does not attempt to infer lineage specific transmission rates, but any future incorporation of lineage specific analyses will require this to be considered. Among the three approaches, the tree only approach detected the earliest uptick in the R_t_ trajectory due to the step-change in transmission rate (*Fig 6*) by a small margin, but all three approaches indicated the increase in a timely manner (within 1 day). The combined approach confidently inferred the time and magnitude of the increase of transmission, in both the infection and R_t_ trajectories (*Figs 5 and 6*). This approach also led to the best RMSE and CRPS scores for the infection and R_t_ trajectories, and a comparable Brier score to the individual approaches.

Overall, the combined-model tended to reduce uncertainty compared to case-only and phylogenetic-only approaches, as observed by narrowing of the HPD intervals of the infection trajectories, while maintaining coverage (*Table 2*). For all three of the main scenarios, the combined approach led to the best R_t_ CRPS and R_t_ trajectory RMSE, and it consistently outperformed one or both of the individual approaches according to our other metrics. There may be some circumstances, however, where either the pure epidemiological or phylodynamic approaches are preferable, such as if one dataset is suspected to be highly biased or incomplete. This points to the benefit of the versatility of the EpiFusion program; while we emphasise the combined inference abilities of EpiFusion, it is possible to run analyses using either case incidence or the phylogenetic tree alone. Furthermore, the program is sufficiently fast for users to test tree only, incidence only, and combined approaches in a reasonable timeframe. It is also theoretically possible to specify the weight of each dataset’s contribution to the inference, allowing further customisation of the combined approach. Going forward, we aim to characterise the implementation and effect of data weighting more thoroughly.

In Fig 7 we explore the effect of increasing transmission and observation noise on the ability of the EpiFusion models to accurately infer R_t_. Currently we do not explicitly model observation noise in the EpiFusion algorithm, however the tree only approach appears particularly robust to even high levels of observation noise. This is possibly due to the extra information provided by branching events in the tree providing a smoothing effect despite noisy sampling rates, and further indicates the possible benefit using phylogenetic data rather than solely case incidence data when estimating R_t_. Interestingly, the Brier Score saw an improvement for all three approaches with increasing transmission noise. We believe that the increased transmission noise resulted in more extreme fluctuations in the R_t_ which provided more signal for the models to distinguish whether R_t_ was less than or greater than 1.0 (*S8 Fig*).

By benchmarking of EpiFusion’s combined model against existing approaches we show that the model can achieve comparable or improved results compared to established epidemiological or phylodynamic tools. For many of the performance metrics used, the difference in scores between all models was marginal, however, EpiFusion led to improved R_t_ RMSE in all scenarios compared all other models (*Table 3).* EpiNow2 proved difficult to parameterise for some scenarios, so it is also possible that an improved parameterisation of the model would result in better estimates. For example, it was not possible to parameterise a step-change in sampling rate in the EpiNow2 model, and the method consequently underperformed in the step-change in sampling scenario.

Finally we examined the performance of EpiFusion using data on the 2014 Ebola outbreak in Sierra Leone. The fact that the most recent common ancestor (MRCA) of the viral phylogeny (March 2014) occurs approximately two months prior to the first sampled case of Ebola in the region (May 2014) allowed modelling of R(t) from an earlier time point than would have been possible using case incidence data alone. We found the model to be sensitive to the sampling parameterisation due to temporal bias in the sampling of genomic sequences compared to the case data, i.e. large fluctuations in the genomic sampling rate of cases over time would sometimes result in particle depletion (a steep drop in the number of particles inferring ‘possible’ trajectories) between particle resampling steps a higher rejection rate of the MCMC algorithm. For this reason, it was necessary to run the model for a larger number of MCMC steps than necessary using simulated data in order to improve the effective sample sizes of model parameters. Similarly, we found that it was necessary to run the particle filter with a greater number of particles to avoid this particle depletion, which also contributed to a slightly longer runtime than the other analyses in this paper. Despite these two caveats we found that R(t) inferred from EpiFusion for this outbreak was similar to that previously obtained in the literature [50,54,55].

Our approach retains some limitations and necessitates some assumptions that provide opportunity for future improvements. As with many models of this type, the model may underperform or exhibit convergence issues if provided with especially biased case incidence or phylogenetic tree data, for example in the early stages of an emerging outbreak where misdiagnosis as other conditions may be common and reported cases may comprise of a combination of autochthonous and imported cases. Thus we advise potential users to exercise discretion in when considering their data inputs. Unlike other phylodynamic approaches such as TimTam, EpiFusion does not estimate phylogenies alongside trajectories, and instead takes single phylogenetic trees as inputs. We aim to better account for phylogenetic uncertainty in the future. However, the computational trade-off of not performing tree inference means that our method may be appropriate for use in rapidly unfolding outbreaks once it has been further validated in a real-time setting, as it is highly scalable to inclusion of trees with thousands of tips. Although not yet optimised for high performance computing or able to take advantage of a GPU, the runtime of EpiFusion generally scales linearly with both tree and epidemic size (*S11 Fig*), making it suitable to analyse very large datasets, which may become more relevant due to the sharp increase in genomic sequencing during the recent COVID-19 pandemic. The model is therefore currently best suited as a post-hoc tool using an MCC tree generated with BEAST [50], or a time-scaled maximum-likelihood phylogeny such as that which can be generated using NextStrain [65].

The lightweight composition of this model provides the opportunity for the future introduction of additional complexity without overtly increasing computational load. This includes the introduction of population structure or vector population dynamics. The separation of the phylogenetic and epidemiological observation models in EpiFusion also lays the foundation for the combination mathematical epidemiological models that previously would have been too complex to integrate into the phylodynamic likelihood with phylogenetic data to jointly model epidemic trajectories.

In conclusion, we propose EpiFusion as a new addition to the small, but growing, number of tools that integrate phylodynamics and epidemiology for the modelling of infectious disease. EpiFusion builds upon the foundation laid by its predecessors to make improvements in computational efficiency, temporal resolution and flexibility.

## Supporting information

S1 Text Information on the importance sampling implementation used within EpiFusion. (DOCX)

S2 Text Pseudocode for the two key EpiFusion algorithms: (1) the MCMC algorithm and (2) the particle filtering algorithm. (DOCX)

S3 Text Details on the model parameterisation for the benchmarking section, where existing Rt modelling methods were used. (DOCX)

S1 Fig The fit of the simulated incidence from the EpiFusion model weekly incidence data as explained in the methods section. The black dots represent case incidence data points *c*_*t*_, which are compared to *ρ*_*interval*_ by the epidemiological observation model. We save the *ρ*_*interval*_ values from the model to facilitate examination of this fit. The coloured lines show the mean *ρ*_*interval*_ values and the shaded regions show HPD intervals of increasing credible mass. Here we show the results of this fit for the combined and case incidence-only approaches in the Scenario Testing section (the tree-only models do not have an epidemiological observation model so this fitting does not take place).
(TIFF)

S2 Fig True infection trajectories, case incidence data, and phylogenetic trees for the step change in sampling (a, b, c) and transmission scenarios (d, e, f) in the Scenario Testing section.
(TIFF)

S3 Fig True infection trajectories, case incidence data, and phylogenetic trees for simulated outbreaks with increasing transmission noise. Transmission noise was simulated in ReMASTER by varying the transmission rate at regular intervals drawn from a Poisson distribution with rate 6 days.
(TIFF)

S4 Fig True infection trajectories, case incidence data, and phylogenetic trees for simulated outbreaks with increasing observation noise. Observation noise was simulated in ReMASTER by varying the sampling rate at intervals of 7 days.
(TIFF)

S5 Fig Publicly available existing MCC tree of Ebola sequences from 2014 obtained from Dellicour et. al (53). The highlighted clade consisting of predominantly Sierra Leone sequences was subsampled for our analysis, and the small Guinea subclades and singleton nodes that represent repeated exports from Sierra Leone were removed. The origin of the highlighted clade was March 20^th^ 2014, which preceded the first case data in Sierra Leone. We therefore modelled the outbreak from this date until the date of the last sampled sequence in the clade (August 4^th^ 2015).
(TIFF)

S6 Fig Weekly confirmed and suspected cases of Ebola in Sierra Leone during the period of investigation obtained from Fang et. al. The first confirmed case was on May 18^th^ 2014, two months after the root of the MCC tree that we used and the beginning of the time period we modelled. For our model, we fit to confirmed cases, but used the suspected cases to help inform our sampling rate priors by indicating what proportion of the true number of infections were being sampled as cases.
(TIFF)

S7 Fig Comparison of EpiFusion and BDSky likelihoods on the same datasets for varying values of (a) beta, (b) gamma and (c) psi around the true values (marked by the blue vertical line). The stochastic and approximate nature of the EpiFusion likelihood means the values are not identical, though they do show good agreement in awarding the true value with the highest likelihood. As the model values of each parameter become further from the true value, the EpiFusion likelihood shows a tendency to drop sharply due to the parameters values implying very unlikely or impossible trajectories. The EpiFusion models appear to demonstrate a marginal overestimation of the sampling parameter psi here, however this was not seen in the simulation based calibration.
(TIFF)

S8 Fig Rt trajectory fits for EpiFusion models on datasets with increasing transmission noise. The true Rt (black line) fluctuates in intervals of ~ 6 days. The row labels (right) indicate the noise level (see Methods ‘Noise Testing’ for more information).
(TIFF)

S9 Fig Rt trajectory fits for EpiFusion models on datasets with increasing observation noise. The real Rt (black line) is smooth with increasing uncertainty in the fits introduced by noisy data, where the sampling rate changed every 7 days. The row labels (right) indicate the noise level (see Methods ‘Noise Testing’ for more information).
(TIFF)

S10 Fig Trajectory fits for a random sample of 60 of the 500 models fitted in the Simulation Based Calibration section. The true trajectory is marked by the black line, with the mean inferred trajectory represented by the green line and the HPD intervals indicated by shaded green regions.
(TIFF)

S11 Fig (a, b, c) Runtime statistics for EpiFusion models with increasing tree size, outbreak size (peak number of individuals infected), and outbreak length (days) using data from the Simulation Based Calibration. Runtime scales linearly with tree size. Runtimes represent the time taken (in minutes) to generate 2000 MCMC samples from EpiFusion on a Macbook Air M3 8-core CPU. EpiFusion has not yet been configured to run on a GPU. (d) Boxplots of the number of effective samples from the posterior generated per minute for the four key EpiFusion particle MCMC variables. Only the initial value of the infection rate beta is shown as beta is fitted as a changing variable over time within the particle filter. According to these times, to yield over 100 effective samples from the posterior for each variable will take approximately 25 minutes.
(TIFF)

S1 Table Summary of the 500 replicate outbreaks modelled (with varying parameters) for the Simulated Based Calibration section. We show characteristics of the datasets: the median epidemic peak (max number of individuals infected at one time); number of cases; and tree size. Next we show `scaled deviated from truth`for gamma, phi and psi parameters. This is calculated as the difference between the model mean and the true value of the parameter, scaled by the true value of the parameter. Finally we show runtime in minutes to generate 2000 MCMC samples.
(XLSX)

S2 Table . ReMASTER parameters for outbreak simulations for the Scenario Testing section. The ‘Main Scenarios’ include the Baseline, Sampling and Transmission. Here constant rates were used for each reaction. In the ‘sampling’ scenario, the rate of sampling was increased 10-fold on day 35. In the transmission scenario, the rate of transmission was increased 3-fold on day 100. For the noise scenarios, either transmission or sampling rates were changed at regular intervals (intervals drawn from a Poisson distribution with rate 6 for the transmission noise, and every 7 days for the observation noise). We added increased noise by drawing interval rate values from distributions with increasing standard deviations.
(XLSX)

S3 Table EpiFusion model parameter priors for each model in the Scenario and Noise Testing section. For the Noise Testing section, the same priors were used for all models.
(XLSX)

S4 Table EpiFusion model results by parameter for each model in the Scenario Testing section. (XLSX)

S5 Table Calculation methods for metrics used to assess model performance. (XLSX)
