# Supplementary material for: EpiFusion: Joint inference of the effective reproduction number by integrating phylodynamic and epidemiological modelling with particle filtering
Source: PLoS Comput Biol. 2024 Nov 11;20(11):e1012528. doi: 10.1371/journal.pcbi.1012528 (PMC11581393; doi:10.1371/journal.pcbi.1012528)
Supplement: S1 Text — (DOCX) [file pcbi.1012528.s001.docx]

# Appendix 1: Importance Sampling Implementation

To prevent the simulation of trajectories that are incompatible with the phylogenetic tree we implement importance sampling. This results in a subtle change to the exact implementation of the process model (*Main Text Eqn 1*) where the terms are adjusted based on the structure of the phylogenetic tree. Specifically, the infection rate, $\beta_{t}$, is first divided into *observed* and *unobserved* infection rates $\beta$*^o^* and $\beta$*^u^* *(Eqns 6&7).* The rate $\gamma$ is adjusted to prevent recovery events that violate the phylogenetic tree structure (i.e., that lead to estimates of the number of infections that are lower than the number of concurrent viral phylogenetic lineages, $l_{t}$), resulting in *allowed* and *forbidden* recovery rates $\gamma^{a}$ and $\gamma^{f}$ *(Eqns 8&9)*.

|  | ${\beta^{o}}_{t}=\beta_{t}\left( \frac{l_{t}\cdot(l_{t}-1)}{I_{t-1}\cdot(I_{t-1}+1)} \right)$ | *(6)* |
| --- | --- | --- |
|  | ${\beta^{u}}_{t}= \beta_{t} - {\beta^{o}}_{t}$ | *(7)* |
|  | ${\gamma^{a}}_{t}= \left\{ \begin{aligned} \gamma_{t} for l_{t}< I_{t}-\gamma_{t}I_{t-1} \\ 0 forl_{t}>= I_{t}-\gamma_{t}I_{t-1} \end{aligned} \right.$ | *(8)* |
|  | ${\gamma^{f}}_{t}= \gamma_{t} - {\gamma^{a}}_{t}$ | *(9)* |

These equations give the model for epidemic trajectory simulation under importance sampling:

|  | $I_{t}=I_{t-1}+ Pois({\beta^{u}}_{t}I_{t-1})-Pois({\gamma^{a}}_{t}I_{t-1})$ | *(10)* |
| --- | --- | --- |

This also necessitates an adjustment of the phylodynamic weight of the particles (*Eqn 3*). Thus, the phylodynamic weight is the product of the conditional probability of the tree given the compartment size $P(g_{t}|I_{t})$ with the ratio of the probability of the trajectory under the process model, $P(I)$, to the probability of the trajectory under the importance distribution $P'(I)$ (*Eqn 11*).

|  | $\omega_{gt}=\frac{P\left( g_{t} \right\vert I_{t})P(I)}{P'(I)}=exp\left( b_{t}log\left( \frac{2\beta_{t}}{I_{t}-1} \right)+s_{t}log\psi_{t}-({\psi_{t}I}_{t-1}+{\beta^{o}}_{t}I_{t-1}+{\gamma^{f}}_{t}I_{t-1}) \right)$ | *(11)* |
| --- | --- | --- |
