# Supplementary material for: EpiFusion: Joint inference of the effective reproduction number by integrating phylodynamic and epidemiological modelling with particle filtering
Source: PLoS Comput Biol. 2024 Nov 11;20(11):e1012528. doi: 10.1371/journal.pcbi.1012528 (PMC11581393; doi:10.1371/journal.pcbi.1012528)
Supplement: S2 Text — (DOCX) [file pcbi.1012528.s002.docx]

# Appendix 2: EpiFusion Pseudocode

### MCMC Algorithm

**Start**

Parse parameters and data from XML input file

**For each MCMC chain:**

**Initialise MCMC:**

**While Particle Filter Likelihood == -Inf:**

Sample parameter set from priors

Run Particle Filtering Algorithm

**For each MCMC step:**

Propose new parameter set

Run Particle Filtering Algorithm

Accept/Reject parameters

**If (step % logging interval) == 0:**

Log parameters

Log infection and beta trajectory

Log current log-likelihood

### Particle Filtering Algorithm

**Start**

Convert gamma psi and/or phi parameters to rates across time for days 0->T

Set each particle beta(0) to value of `initialbeta` parameter

Set each particle I(0) to 1

Set each particle positiveTests to 0

**For each day ‘t’ in 0->T:**

**For each particle:**

Sample new beta(t) from previous day’s beta

Get infection, recovery and sampling `propensities`*

**If phylogenetic tree is provided:**

Adjust beta and gamma propensities according to Eqn 6-9

Sample new infections and recoveries

Set state I(t) according to Eqn 10

**If case incidence data is provided:**

Sample positiveTests from I using rate phi

Increment particle positiveTests

Increment phylodynamic likelihood using Eqn 3

**If t has a case incidence data point:**

Increment epidemiological likelihood using Eqn 2

Set positiveTests to 0

**Else:**

Sample new infections and recoveries

Set state I(t) according to Eqn 1

**If t is a ‘resampling day’:**

**If phylogenetic tree is provided:**

Rescale phylogenetic likelihoods and get phylo weight

**If case incidence is provided:**

Rescale epi likelihoods and get epi weight

Get combined weight using Eqn 4

Resample particles according to combined weight

Increment particle filter likelihood using Eqn 5

**Else:**

Resample particles according to phylo weight

Increment particle filter likelihood using Eqn 5

**Else:**

Resample epidemiological likelihoods and get epi weight

Resample particles according to epi weight

Increment particle filter likelihood using Eqn 5

Set weights and likelihoods back to 0

**Return** particle filter likelihood

* Propensities: rate(t) multiplied by current number of people infected I(t).
