# Supplementary material for: EpiFusion: Joint inference of the effective reproduction number by integrating phylodynamic and epidemiological modelling with particle filtering
Source: PLoS Comput Biol. 2024 Nov 11;20(11):e1012528. doi: 10.1371/journal.pcbi.1012528 (PMC11581393; doi:10.1371/journal.pcbi.1012528)
Supplement: S3 Text — (DOCX) [file pcbi.1012528.s003.docx]

# Appendix 3: Benchmarking Model Parameterisation

### EpiNow2 Parameterisation

EpiNow2 (v1.5.1) [51] was used to analyse the simulated weekly case incidence for each scenario. The sampling delay was parameterised using the delay_opts() function with a distribution identical to the parameters under which the data was simulated. The generation time was parameterised using a probability mass function of the generation time using the known values from the data simulation process, and input to the epinow() function using generation_time_opts(). The epinow() function was used with default parameters, and a horizon of 0. Code used for this section is included in the project Github repository in the ‘Benchmarking’’ folder.

### BDSky Parameterisation

Packages and software used: BDSKY 1.5.0 package [14] with BEAST 2.7.6 [65]. XML files for these runs and log files of the results are included in the project GitHub repository in the ‘Benchmarking’ folder. Convergence was assessed using Tracer (v1.7.2).

### TimTam Parameterisation

Packages and software used: TimTam 0.4.0 [30] package with BEAST. 2.7.6 [65]. XML files for these runs and log files of the results are included in the project Github repository in the ‘Benchmarking’ folder. The analyses loosely followed the following tutorials on the TimTam Github Wiki: ‘Variable Parameters for an SIR Epidemic I’ and ‘Variable Parameters for an SIR Epidemic II’. Convergence was assessed using Tracer (v1.7.2).
