# Supplementary figures and images for: EpiFusion: Joint inference of the effective reproduction number by integrating phylodynamic and epidemiological modelling with particle filtering
Source: PLoS Comput Biol. 2024 Nov 11;20(11):e1012528. doi: 10.1371/journal.pcbi.1012528 (PMC11581393; doi:10.1371/journal.pcbi.1012528)

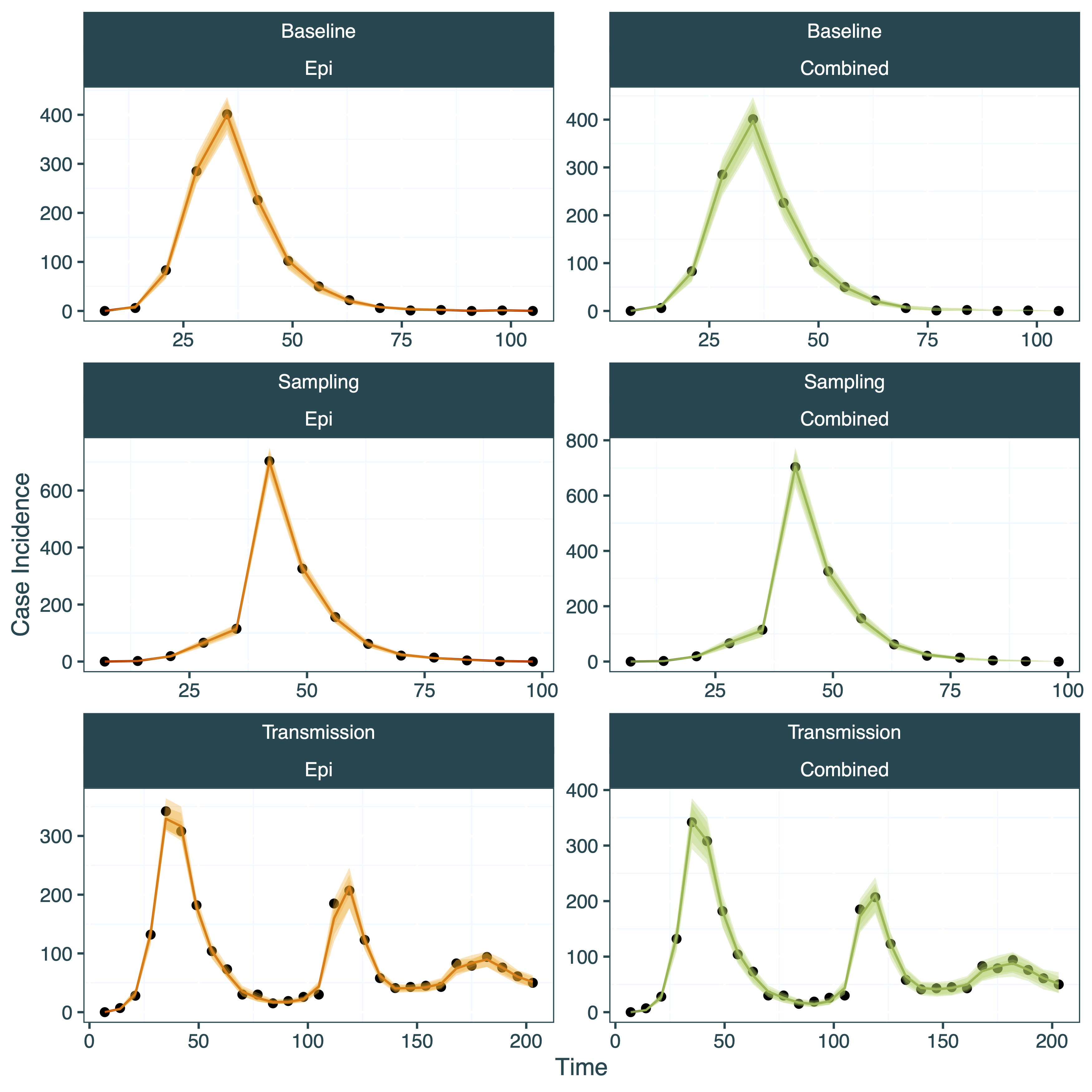

Supplement: S1 Fig — The black dots represent case incidence data points ct, which are compared to ρinterval by the epidemiological observation model. We save the ρinterval values from the model to facilitate examination of this fit. The coloured lines show the mean ρinterval values and the shaded regions show HPD intervals of increasing credible mass. Here we show the results of this fit for the combined and case incidence-only approaches in the Scenario Testing section (the tree-only models do not have an epidemiological observation model so this fitting does not take place). (TIFF) [file pcbi.1012528.s004.tiff]

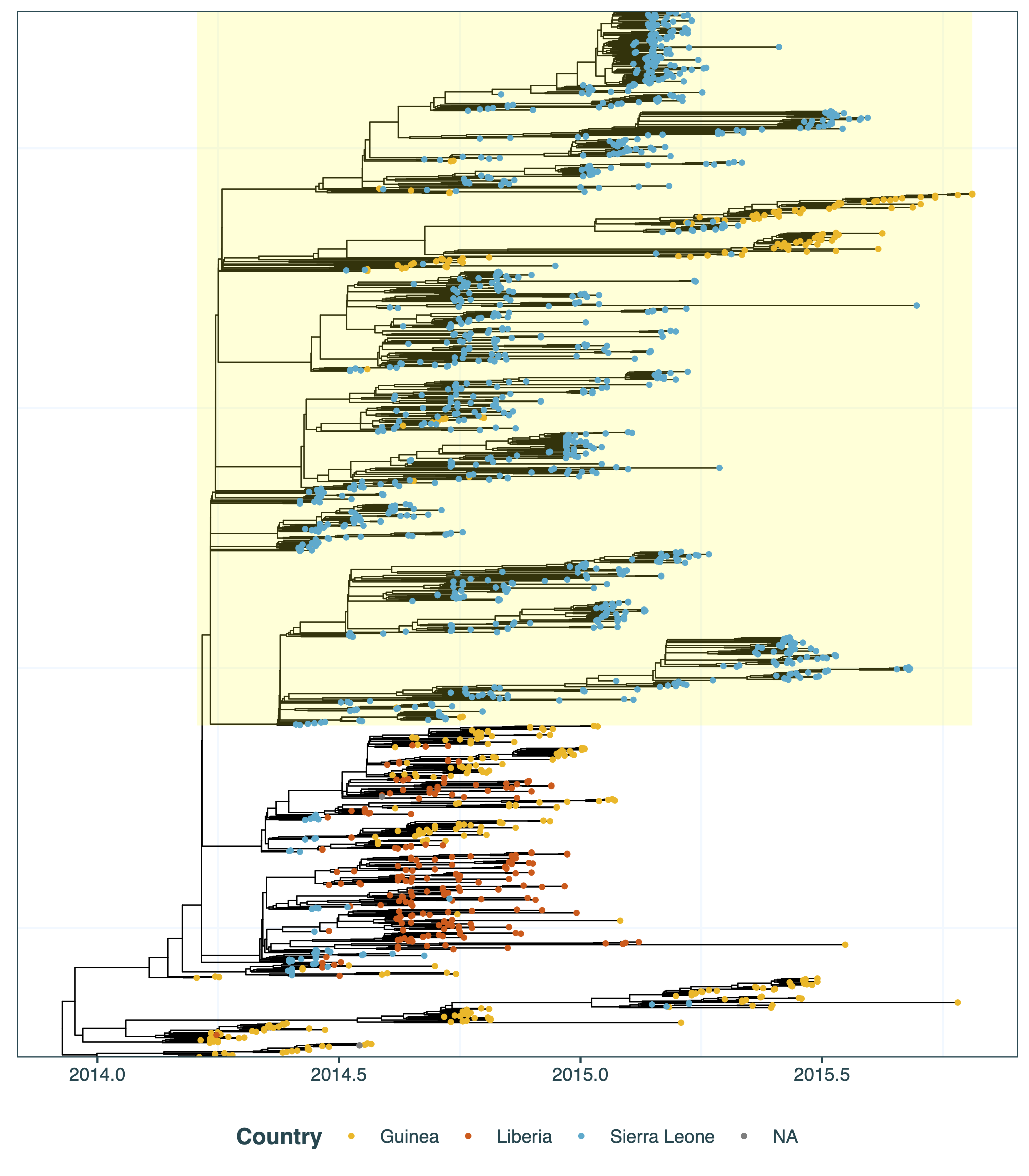

Supplement: S5 Fig — The highlighted clade consisting of predominantly Sierra Leone sequences was subsampled for our analysis, and the small Guinea subclades and singleton nodes that represent repeated exports from Sierra Leone were removed. The origin of the highlighted clade was March 20th 2014, which preceded the first case data in Sierra Leone. We therefore modelled the outbreak from this date until the date of the last sampled sequence in the clade (August 4th 2015). (TIFF) [file pcbi.1012528.s008.tiff]

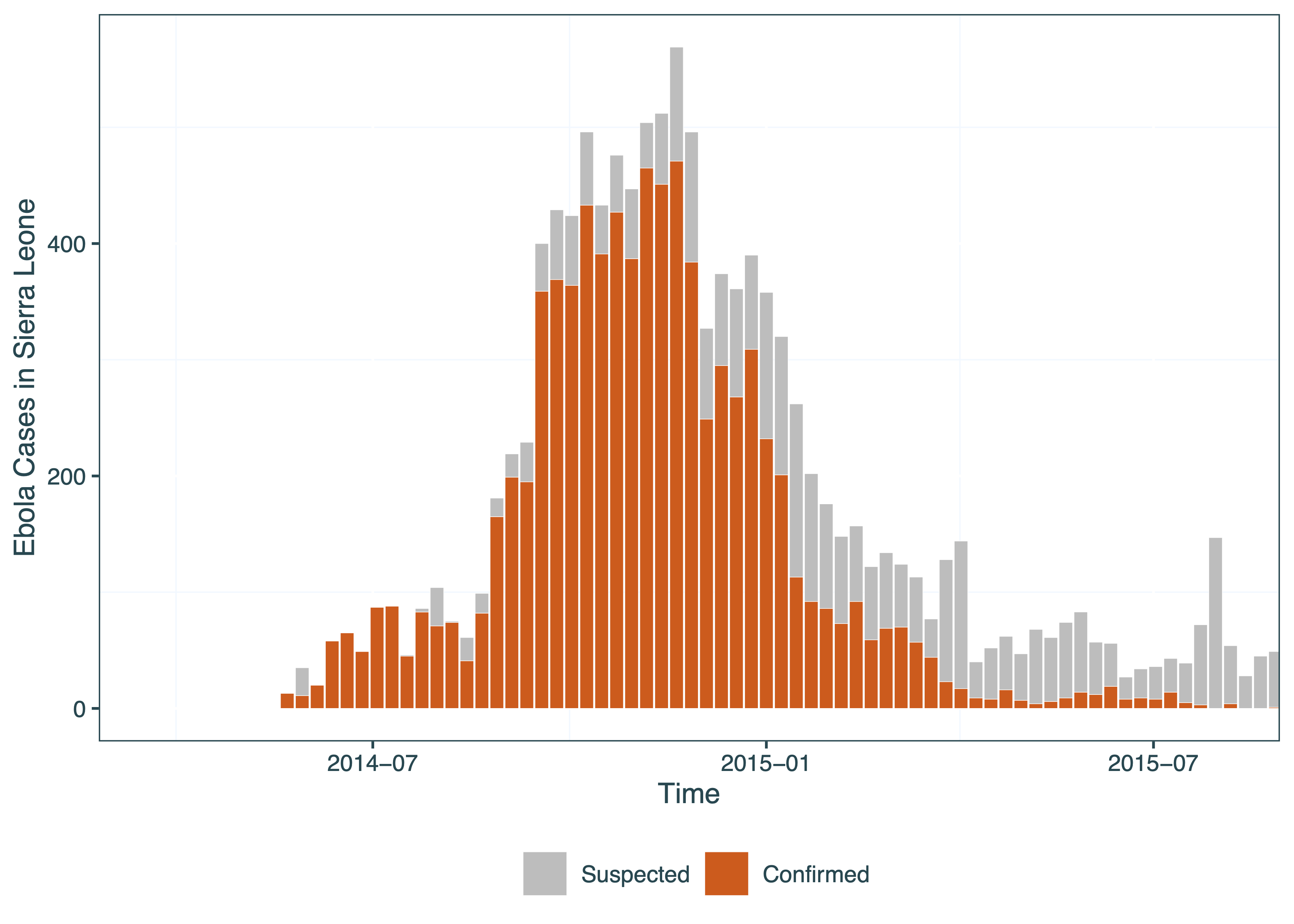

Supplement: S6 Fig — The first confirmed case was on May 18th 2014, two months after the root of the MCC tree that we used and the beginning of the time period we modelled. For our model, we fit to confirmed cases, but used the suspected cases to help inform our sampling rate priors by indicating what proportion of the true number of infections were being sampled as cases. (TIFF) [file pcbi.1012528.s009.tiff]

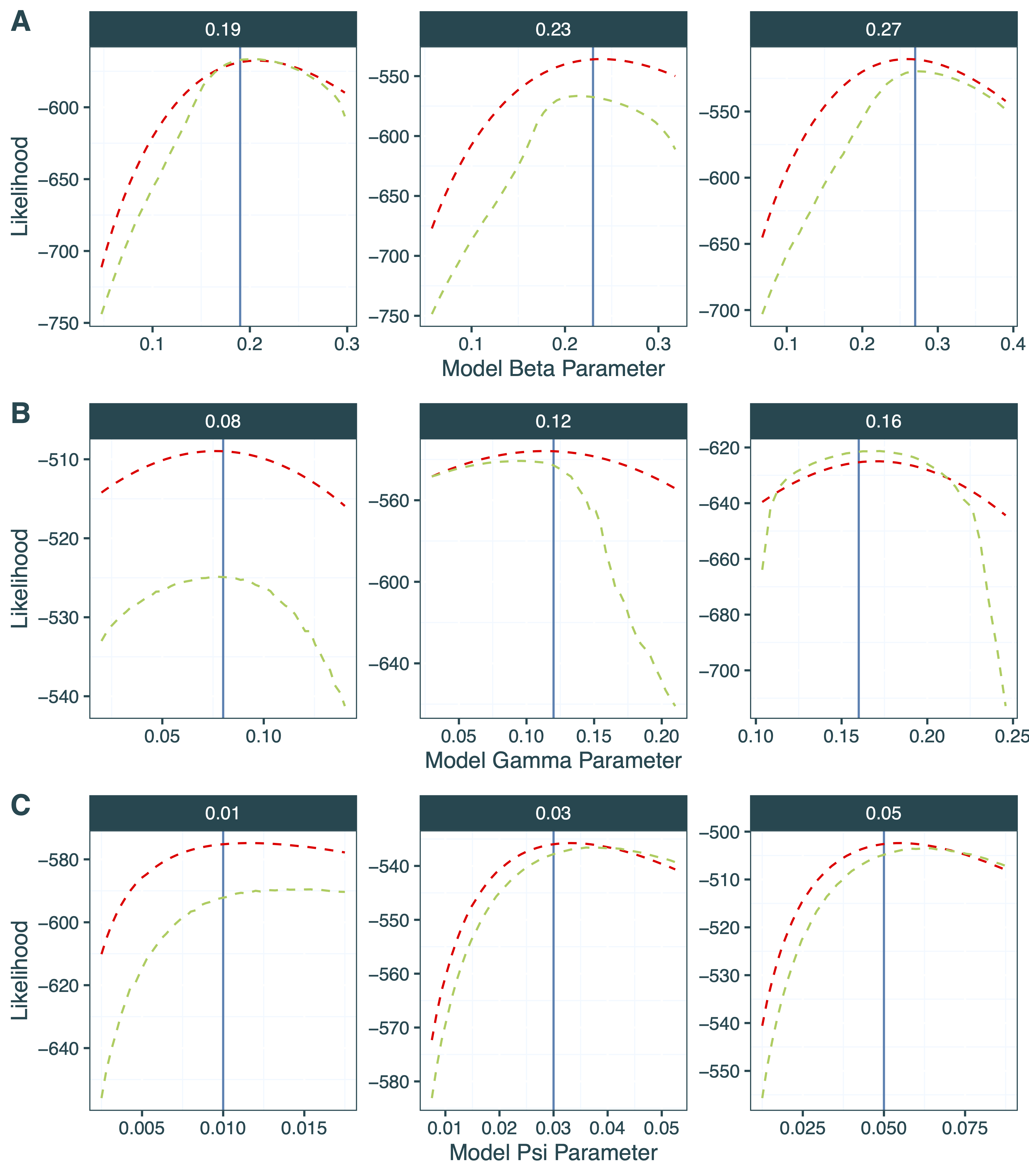

Supplement: S7 Fig — Comparison of EpiFusion and BDSky likelihoods on the same datasets for varying values of (a) beta, (b) gamma and (c) psi around the true values (marked by the blue vertical line). The stochastic and approximate nature of the EpiFusion likelihood means the values are not identical, though they do show good agreement in awarding the true value with the highest likelihood. As the model values of each parameter become further from the true value, the EpiFusion likelihood shows a tendency to drop sharply due to the parameters values implying very unlikely or impossible trajectories. The EpiFusion models appear to demonstrate a marginal overestimation of the sampling parameter psi here, however this was not seen in the simulation based calibration. (TIFF) [file pcbi.1012528.s010.tiff]

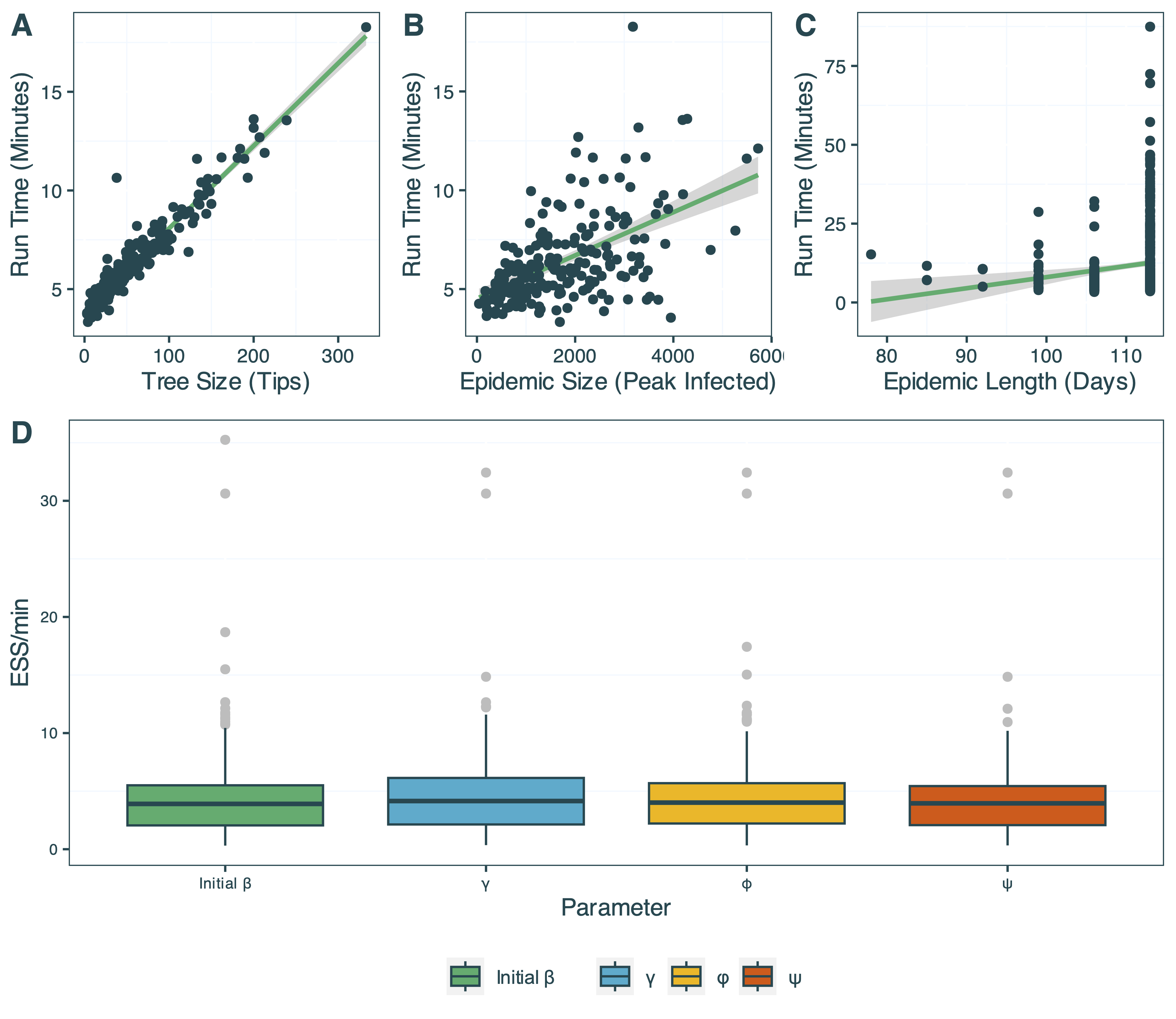

Supplement: S11 Fig — (a, b, c) Runtime statistics for EpiFusion models with increasing tree size, outbreak size (peak number of individuals infected), and outbreak length (days) using data from the Simulation Based Calibration. Runtime scales linearly with tree size. Runtimes represent the time taken (in minutes) to generate 2000 MCMC samples from EpiFusion on a Macbook Air M3 8-core CPU. EpiFusion has not yet been configured to run on a GPU. (d) Boxplots of the number of effective samples from the posterior generated per minute for the four key EpiFusion particle MCMC variables. Only the initial value of the infection rate beta is shown as beta is fitted as a changing variable over time within the particle filter. According to these times, to yield over 100 effective samples from the posterior for each variable will take approximately 25 minutes. (TIFF) [file pcbi.1012528.s014.tiff]
